# Targeting CREB3L2-mediated lipid metabolism overcomes lenvatinib resistance and attenuates the progression of hepatocellular carcinoma

**DOI:** 10.1038/s41419-025-08250-3

**Published:** 2025-11-24

**Authors:** Shiguang Yang, Shaoqing Liu, Jie Li, Shengwei Mao, Qifeng Yu, Xuhui Zhao, Xiaoling Wu, Jiafeng Chen, Yichao Bu, Weiren Liu, Yuan Fang, Yinghong Shi

**Affiliations:** 1 Department of Hepatobiliary and Pancreatic Surgery, Key Laboratory of Whole-Period Monitoring and Precise Intervention of Digestive Cancer, Minhang Hospital, Fudan University, Shanghai, China; 2 Department of Liver Surgery, Key Laboratory of Carcinogenesis and Cancer Invasion, Liver Cancer Institute, Zhongshan Hospital, Fudan University, Shanghai, China; 3 Department of Breast Surgery, Department of Emergency Medicine, The First Affiliated Hospital, Zhengzhou University, Zhengzhou, China; 4 National Medical Center & National Clinical Research Center for Interventional Medicine, Liver Cancer Institute, Zhongshan Hospital, Fudan University, Shanghai, China; 5 Department of Hepatobiliary Surgery, Zhoushan Hospital, Zhejiang, China

**Keywords:** Cancer therapy, Tumour biomarkers

## Abstract

When hepatocellular carcinoma (HCC) cells exhibit malignant biological behaviors, lipid metabolic reprogramming occurs concomitantly. Thus, identifying regulators of metabolic reprogramming offers new potential targets for therapy. In this study, we investigated the mechanisms by which Cyclic adenosine monophosphate-responsive element binding protein 3-like 2(CREB3L2) influences HCC progression and contributes to lenvatinib resistance through modulation of lipid metabolism. Up-regulated expression of CREB3L2 was observed in numerous HCC cohorts and associated with poor survival prognosis of patients. Furthermore, CREB3L2 could facilitate the proliferation and metastatic capacity of HCC cells both in vitro and in vivo. It was found that CREB3L2 influences the proliferation and metastasis of HCC cells by up-regulating sterol regulatory element binding protein 1 (SREBP1), a vital regulatory factor of lipid synthesis for fatty acid production. Additionally, CREB3L2 enhances SREBP1 protein expression and stability through increased acetylation mediated by histone acetyltransferase-1(HAT1). Importantly, targeting CREB3L2 in combination with lenvatinib significantly reduced lenvatinib resistance, inhibiting the progression of CREB3L2 high-expressing HCC tumors. These findings suggest that the CREB3L2/HAT1/SREBP1 regulatory axis drives lenvatinib resistance and HCC progression by impacting lipid metabolism. Targeting CREB3L2 alongside lenvatinib improves the efficacy of treating HCC.

## Introduction

Primary liver cancer is a prevalent form of cancer globally, ranking among the top three causes of death in cancer patients. Among them, hepatocellular carcinoma (HCC) is the most common pathological type, accounting for 85% of cases [1]. After undergoing surgical treatment for early-stage HCC, despite the potential administration of various adjuvant therapies, the survival rate for these patients remains at approximately 30% due to the occurrence of tumor recurrence and metastasis [2, 3]. Hence, it is crucial to clarify the underlying mechanisms of HCC malignant biological behaviors and identify effective therapeutic targets.

The Cyclic adenosine monophosphate-responsive element binding protein 3 (CREB3), distinguished by its leucine zipper motif and ubiquitous expression in humans, consists of DNA-binding proteins. This family comprises CREB3 and its four isoforms, namely CREB3L1, CREB3L2, CREB3L3, and CREB3L4, which play pivotal roles in regulating cellular protein metabolism through the unfolded protein response [4, 5]. It is reported that CREB3L2 protein can be hydrolyzed by intramembrane proteins to produce cytosolic CREB3L2, which promotes thyroid carcinoma cell proliferation and inhibits camp response transcription [6]. Meanwhile, dysregulation of CREB3L2 is strongly linked to malignant glioma, prostate cancer, and soft tissue sarcoma [7–9]. Although a mass of studies indicate the significant role of CREB3L2 in cancer, its involvement in HCC requires further exploration in the long term.

Metabolic reprogramming is gradually recognized as a fundamental characteristic in the process of cancer development [10]. In addition to alterations in glucose and glutamine metabolism, the significance of lipid metabolic reprogramming in cancer progression is also pivotal [11]. Most normal cells prefer to utilize exogenous fatty acids, whereas cancer cells exhibit a preference for de novo lipid synthesis, thereby promoting energy storage and malignant proliferation [12–16]. Accumulating evidence supports that de novo lipid synthesis plays a crucial role in driving the progression of HCC [17, 18]. However, the mechanisms responsible for abnormal lipid metabolism in human HCC have not been fully elucidated.

In this research, we scientifically investigated the effect of CREB3L2 in reprogramming lipid metabolism of HCC cells. This paper represents the first investigation into the expression level and function of CREB3L2 in HCC, revealing its impact on HCC cells proliferation and metastasis through modulation of lipid synthesis. Mechanistically, CREB3L2 preserves the stability of SREBP1 protein by suppressing its ubiquitination, with histone acetyltransferase 1 (HAT1)-mediated acetylation enhancing this effect. Notably, the down-regulation of CREB3L2 enhanced the treatment effect of lenvatinib in HCC both in vitro and in vivo. These findings offer new insights into the mechanisms of HCC progression and the identification of potential targets for overcoming lenvatinib resistance, providing a potential strategy for treating specific types of HCC.

## Materials and methods

### Clinical patient samples

This study included 206 patients with HCC, who were drawn from two separate cohorts at Zhongshan Hospital of Fudan University (Shanghai, China). Cohort 1 included postoperative tissue samples from 30 HCC patients who were hospitalized between December 2019 and March 2022. Cohort 2 consisted of tissue microarrays collected from 176 tumor patients who visited the hospital between January 2009 and January 2010. None of these patients received any form of neoadjuvant therapy prior to surgery.

### Cell culture

Human HCC cell lines MHCC97H, MHCCLM3, Huh-7, PLC/PRF/5, Hep-3B and human normal hepatocytes L-02 were purchased from the cell bank of Chinese Academy of Sciences. They are free of mycoplasma and have been identified and certified by short tandem repeat. All culture media used for the cells were supplemented with 10% fetal bovine serum and 1% penicillin-streptomycin.

### Animal experiments

For subcutaneous xenografts, control cells, CREB3L2-overexpressing Huh7 cells, or CREB3L2-knockdown 97H cells (2 × 10⁶) were injected into nude mice. Lenvatinib (10 mg/kg) was administered intragastrically starting 1 week post-inoculation. Tumor volume (length × width²/2) was measured every 5 days from day 5, and tumor weight was assessed after euthanasia at 25 days.

For lung metastasis modeling, the same cell groups (2 × 10⁶) were injected via tail vein. Lenvatinib (10 mg/kg) was administered 2 weeks post-injection. Mice were necropsied after 7–8 weeks, with metastatic nodules quantified via HE staining.

### Statistical analysis

Each independent experiment was repeated at least three times, and the results are presented as mean ± SD, either the *T* test or ANOVA was used to analyze the experimental data,with *P* < 0.05 indicates that the difference in component values is statistically significant.

## Results

### CREB3L2 is upregulated and associates with poor outcomes in HCC

**Fig. 1:**
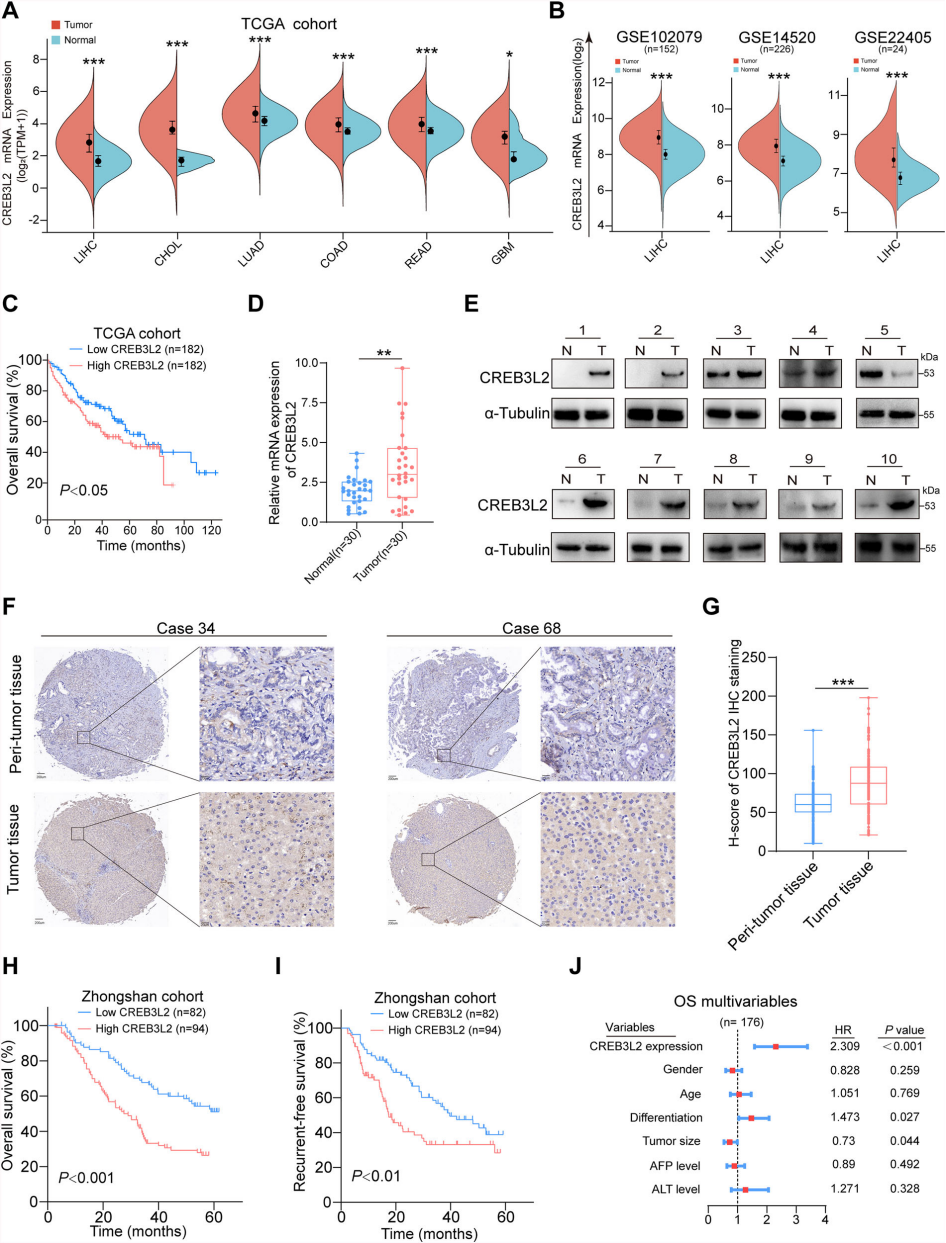
CREB3L2 is upregulated and associates with poor outcomes in HCC. **A** Relative expression profiles of CREB3L2 across multiple tumor types with liver hepatocellular carcinoma (LIHC), cholangiocarcinoma (CHOL), lung adenocarcinomastomach (LUAD), colon adenocarcinoma (COAD), rectum adenocarcinoma(READ), and glioblastoma multiformebased (GBM) on the TCGA database. **B** The expression profiles of CREB3L2 within three patient-specific datasets of HCC sourced from the GEO database (sample identifiers GSE102079, GSE14520, and GSE22405). **C** Survival prognosis curves of HCC patients with different CREB3L2 expression levels. **D** The relative expression levels of CREB3L2 mRNA within 30 pairs of HCC tissues and their adjacent non-neoplastic counterparts. **E** The expression profile of CREB3L2 protein in 10 pairs of liver cancer tissues and their paired adjacent tissues. **F** Typical immunohistochemical staining images illustrate the distinctions in CREB3L2 expression levels between hepatocellular carcinoma (HCC) tissues and their peritumoral counterparts. **G** IHC staining scores for the expression of CREB3L2 protein in 176 sets of paired tumor tissues. **H**, **I** Prognostic curves showing OS and RFS of patients in Cohort 2. **J** Multivariate Cox analysis of OS clinical prognostic parameters. *P < 0.05; **P < 0.01; ***P < 0.001.

To determine the expression pattern of CREB3L2 in human malignancies, we conducted a comparison of mRNA levels across 23 different tumor types using data from TCGA database. Public RNA sequencing data indicate that CREB3L2 is upregulated in a variety of tumors, including HCC (Fig. 1A and Supplementary Fig. S1A), while HCC patients with high CREB3L2 expression demonstrated a lower overall survival (OS) rate compared to those with lower expression levels (Fig. 1C). We further examined the clinical relevance of CREB3L2 expression by analyzing three separate HCC patient dataset from the GEO (Fig. 1B), the results also verify that the expression of CREB3L2 is sustainedly markedly upregulated in HCC tumor samples compared to adjacent non-tumor tissues.

To validate the mRNA level of CREB3L2 in the public dataset, we performed qRT-PCR assay on 30 pairs of HCC samples (Cohort 1) and showed that 56.66% (17/30) of HCC patients had at least two-fold upregulation of CREB3L2 (Fig. 1D). Next, we examined CREB3L2 expression in HCC tissues at the protein level, 10 pairs of tissues were randomly selected from Cohort 1 for Western blotting, among the 9 pairs of samples, the expression level of CREB3L2 was higher in tumor tissues, with only one pair showing relatively lower expression in the tumor (Fig. 1E).

To further investigate whether CREB3L2 expression is associated with the clinicopathologic features or long-term survival of patients with HCC, immunohistochemistry was performed on tissue microarrays to detect CREB3L2 expression in 176 patients with HCC (Cohort 2). Consistent with our previous experimental findings, the CREB3L2 level exhibited a significant increase in tumor tissues compared to the paired peritumoral tissues (Fig. 1F, G). In addition, higher CREB3L2 levels were significantly associated with tumor stage progression in patients, but not with other indicators such as patient age, gender, tumor size, etc (Supplementary Table 1). Meanwhile, patients with high CREB3L2 expression tended to exhibite worse OS and recurrence-free survival (RFS) compared to patients with low CREB3L2 expression (Fig. 1H, I). Furthermore, Cox multivariate proportional hazards modeling also showed that aberrant expression of CREB3L2 was an independent prognostic factor for poorer OS and worse RFS (Fig. 1J and Supplementary Fig. S1B). These results above manifest that up-regulated CREB3L2 expression may contribute to the malignant progression of HCC.

### CREB3L2 facilitates HCC cell proliferation and metastasis in vitro and in vivo

**Fig. 2:**
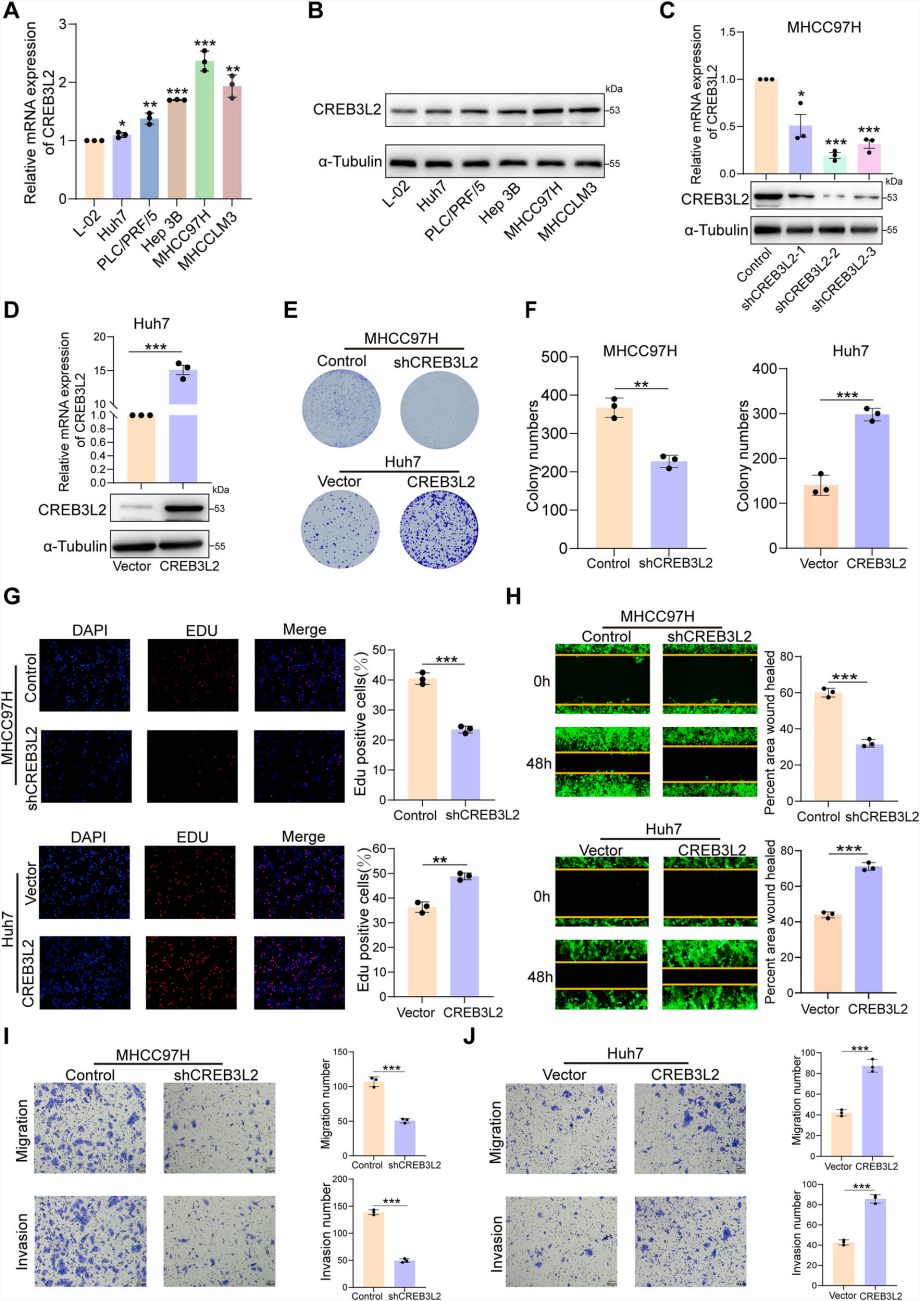
CREB3L2 facilitates HCC cell proliferation and metastasis in vitro. **A**, **B** The expression patterns of CREB3L2 at the mRNA and protein levels in hepatocellular carcinoma cell lines and normal hepatocyte cell lines. **C**, **D** The transfection efficiency of knockdown and overexpression of CREB3L2 in 97H and Huh7 cells. **E–G** Colony formation and EDU assays were implemented following knockdown or overexpression of CREB3L2 in 97H and Huh7 cells. **H–J** After knockdown or overexpression of CREB3L2, scratch wound healing and transwell assays were used to assess the migration and invasion abilities of HCC cells. *P < 0.05; **P < 0.01; ***P < 0.001.

To determine the potential functions and roles of CREB3L2 in HCC, we examined the expression of CREB3L2 in five HCC cell lines by qRT-PCR and Western blotting. The protein and mRNA of CREB3L2 were upregulated in all tested HCC cell lines compared to the normal hepatocyte line L-02 (Fig. 2A, B). For subsequent cell function experiments, we selected the 97H and LM3 cell lines, which exhibit relatively high expression levels of CREB3L2, to construct knockdown cell models. In contrast, we chose the Huh7 and PLC cell lines, characterized by relatively low expression of CREB3L2, to establish overexpression cell models (Fig. 2C, D, Supplementary Fig. S2A, B).

Within the specifically selected HCC cell lines, the colony formation assay demonstrated that alterations in the expression level of CREB3L2 exerted a noteworthy impact on the proliferative capacity of tumor cells (Fig. 2E, F, Supplementary Fig. S2C). Furthermore, we continued to investigate the effect of CREB3L2 on the proliferative capacity of HCC cells using the Edu assay. The results were consistent with those of the clone formation assay: knockdown of CREB3L2 inhibited the proliferative ability of the 97H and LM3, while overexpression of CREB3L2 significantly enhanced the proliferative ability of the Huh7 and PLC (Fig. 2G and Supplementary Fig. S2D). In addition, wound healing assays showed that knockdown of CREB3L2 in 97H and LM3 cells impeded their migratory ability, whereas overexpression of CREB3L2 significantly increased the migratory ability of Huh7 and PLC cells compared to corresponding control cells (Fig. 2H and Supplementary Fig. S2E). Meanwhile, transwell assays also confirmed that dysregulation of CREB3L2 could affect the migration and invasion ability of respective HCC cells (Fig. 2I, J, Supplementary Fig. S2F, G).

**Fig. 3:**
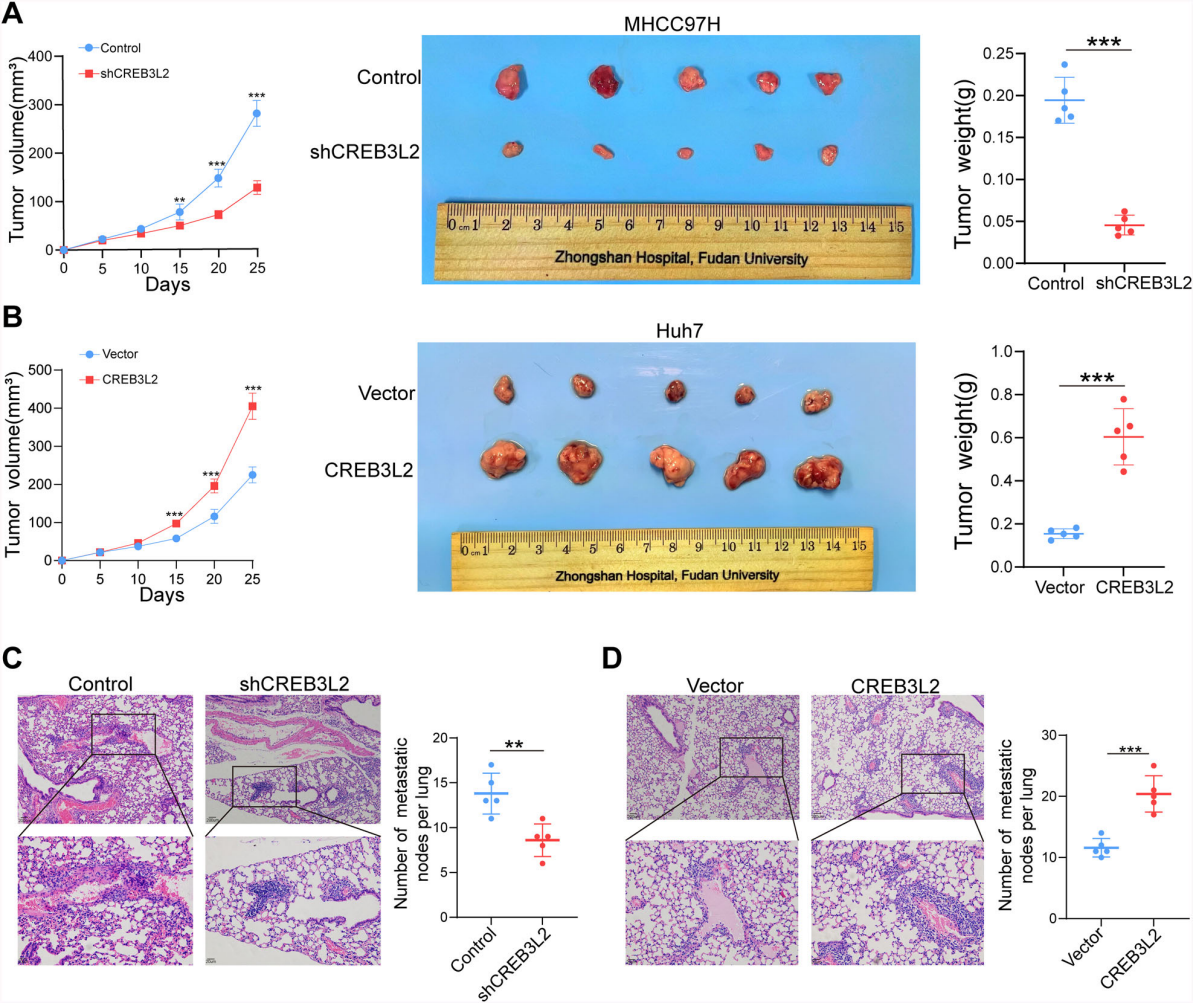
CREB3L2 facilitates HCC cell proliferation and metastasis in vivo. **A**, **B** Subcutaneous tumor volume and weight changes were evaluated in mice after manipulating CREB3L2 expression (knockdown or overexpression). **C**, **D** The effects of CREB3L2 knockdown or overexpression on the nude mice model of pulmonary metastasis. *P < 0.05; **P < 0.01; ***P < 0.001.

Further, we used subcutaneous tumor and tail vein metastasis models in nude mice to assess whether CREB3L2 has the same role in vivo. In contrast to the control group, the knockdown of CREB3L2 elicited a marked deceleration of the tumor growth rate and a substantial reduction in tumor weight (Fig. 3A). Conversely, an elevation in the expression level of CREB3L2 was accompanied by a concomitant increase in both the tumor growth rate and tumor dimensions (Fig. 3B), with corresponding changes in the number of lung metastatic nodules in mice based on the knockdown or overexpression of CREB3L2 (Fig. 3C, D). Collectively, these results indicate that CREB3L2 facilitates the proliferation and metastasis of HCC cells both in vitro and in vivo.

### CREB3L2 modulates fatty acid metabolism pathways

**Fig. 4:**
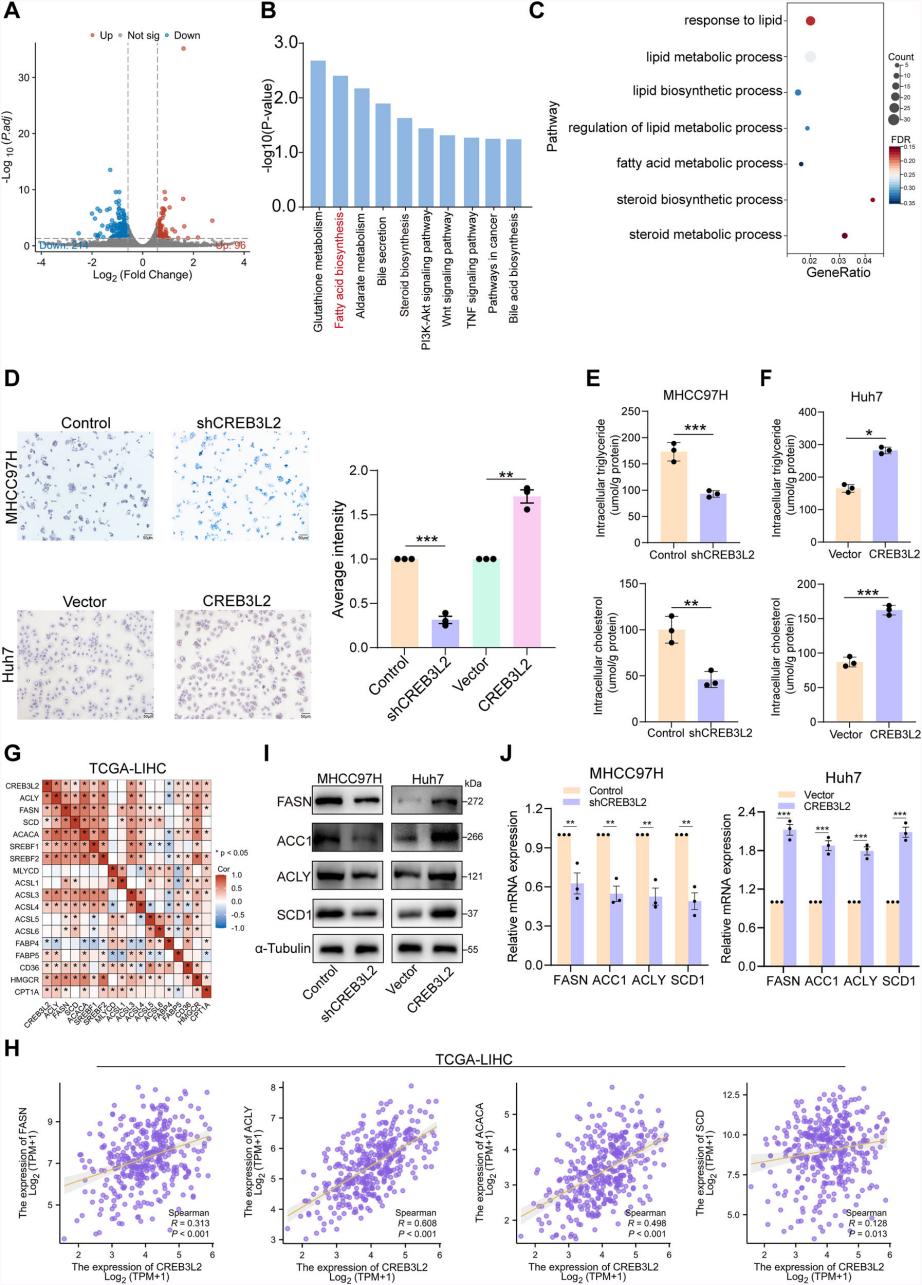
CREB3L2 modulates fatty acid metabolism pathways. **A** Volcano plot showing differentially expressed genes in 97H cells comparing shNC and shCREB3L2 groups. **B**, **C** Differentially expressed genes were conducted to KEGG pathway analysis and GSEA enrichment analysis. **D** Modulation of oleic acid levels in 97H and Huh7 cells upon CREB3L2 knockdown or overexpression. **E**, **F** Determine the levels of triglycerides and cholesterol in 97H and Huh7 cells. **G** Correlation analysis reveals the relationship between CREB3L2 and the expression of lipid metabolism regulatory molecules. **H** Correlation analysis of CREB3L2 expression with the expression of FASN, ACC1, ACLY, and SCD1 in the TCGA database. **I**, **J** After knocking down or overexpressing CREB3L2, assess the alteration of FASN, ACC1, ACLY, and SCD1. *P < 0.05; **P < 0.01; ***P < 0.001.

To investigate CREB3L2-dependent transcriptomic changes and downstream regulatory targets, we performed genome-wide RNA sequencing of MHCC97H cells with CREB3L2 knockdown. A total of 310 genes were differentially expressed after knockdown of CREB3L2 (|fold change| ≥ 2, *P* < 0.05) (Fig. 4A). Subsequently, we found significant changes in fatty acid metabolic pathways by Kyoto Encyclopedia of Genes and Genomes (KEGG) and Gene Ontology enrichment analysis, suggesting that CREB3L2 may play a crucial role in the regulation of fatty acid metabolism (Fig. 4B, C). Oil red staining revealed that dysregulation of CREB3L2 in Huh7 and 97H cells had a significant impact on cellular lipid accumulation (Fig. 4D). Furthermore, the quantification of intracellular triglyceride and cholesterol levels was accomplished via detection kits. Findings indicated that, in comparison to control cells, there was a marked reduction in the intracellular triglyceride and cholesterol levels within CREB3L2-silenced cells, while an increase was observed in cells overexpressing CREB3L2 (Fig. 4E, F).

Next, we sought to explore the mechanisms by which CREB3L2 regulates aberrant lipid metabolism in HCC cells, including increased rate of lipid synthesis, decreased catabolism, and increased fatty acid uptake, all of which contribute to increased cellular lipid content. Therefore,preliminary analysis of the correlation between CREB3L2 and the expression of key molecules of lipid metabolism in HCC tissues by RNA-seq data from HCC in TCGA (Fig. 4G).In addition, Pearson correlation analysis showed a significant correlation between the expression level of CREB3L2 and the fatty acid synthases ACLY, ACC1, FASN and SCD (Fig. 4H). Consistently, the knockdown of CREB3L2 led to a marked inhibition of the expression levels of the aforementioned fatty acid synthesizing enzymes in 97H cells, whereas overexpression of CREB3L2 exerted an impact on their expression levels in Huh7 cells (Fig. 4I, J). Moreover, the expression of key fatty acid oxidation genes (CPT1A and ACOX1) remained unchanged after CREB3L2 knockdown or overexpression (Fig. S3A, B). Based on these findings, it is reasonable to hypothesize that CREB3L2 may impact alterations in cellular lipid metabolism by promoting the expression of key enzymes involved in fatty acid synthesis.

### CREB3L2 attenuates ubiquitinated degradation of SREBP1 protein by enhancing HAT1-mediated acetylation

**Fig. 5:**
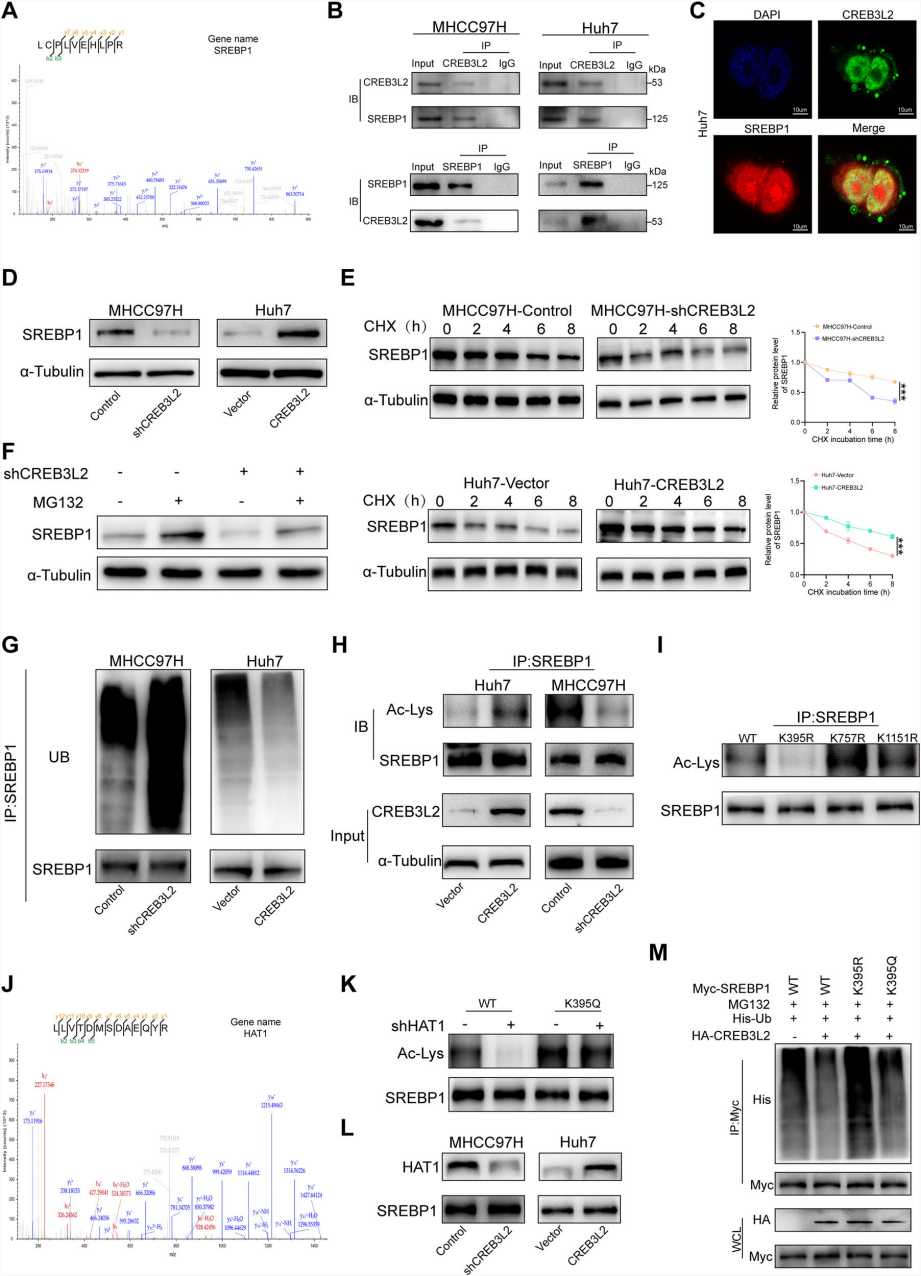
CREB3L2 attenuates ubiquitinated degradation of SREBP1 protein by enhancing HAT1-mediated acetylation. **A** Mass spectrometry analysis of a peptide derived from Flag-CREB3L2 immunoprecipitates to show the potential interaction between SREBP1 and CREB3L2. **B**, **C** Co-IP and the immunofluorescence confocal microscopy demonstrated the colocalization of CREB3L2 and SREBP1 in Huh7 cells. **D** The effect of CREB3L2 on SREBP1 protein levels in HCC cells. **E** Western blot analysis of the effect of CREB3L2 on the half-life of SREBP1 in HCC cells treated with cycloheximide (CHX) for specified time periods. **F** The protein expression level of SREBP1 in 97H-shCREB3L2 cells in the presence of MG132. **G** The effect of knockdown or overexpression of CREB3L2 on the ubiquitination level of SREBP1 in HCC cells. **H** The effect of CREB3L2 expression levels on the acetylation levels of SREBP1 in HCC cells. **I** Observation of changes in SREBP1 acetylation levels after transfection with plasmids containing site-directed mutations. **J** Mass spectrometry analysis of the acetyltransferase HAT1 potentially interacting with SREBP1. **K** The effect of downregulating HAT1 or transfecting acetylation site mutation plasmids on the acetylation levels of SREBP1. **L** The effect of knockdown or overexpression of CREB3L2 on the protein expression level of HAT1. **M** Detection of SREBP1 ubiquitination in Huh7 cells after co-transfection with His-Ub, HA-CREB3L2, Myc-SREBP1 WT, Myc-SREBP1-K395R and Myc-SREBP1-K395Q and treatment with MG132. *P < 0.05; **P < 0.01; ***P < 0.001.

Fatty acid synthase is normally activated in trans by two transcription factors, carbohydrate-responsive element-binding protein and sterol-regulatory element-binding proteins (SREBP). To further investigate the molecular mechanism by which CREB3L2 affects fatty acid synthase, we performed liquid chromatography-tandem mass spectrometry analysis(LC-MS) and identified SREBP1 as a potential CREB3L2-interacting protein (Fig. 5A). Co-immunoprecipitation and immunofluorescence analysis confirmed the interaction and co-localization of CREB3L2 with SREBP1 in HCC cells (Fig. 5B, C). Then, we evaluated the impact of CREB3L2 on SREBP1 expression in HCC cells, and neither the knockdown nor the overexpression of CREB3L2 exerted an influence on the SREBP1 mRNA level, but it did affect SREBP1 expression at the protein level (Fig. 5D and Supplementary Fig. S4A), suggesting that CREB3L2 may regulate the post-translational modification of SREBP1. Furthermore, we treated HCC cells with cycloheximide(CHX) at various specific time points, our results demonstrated that the rate of degradation of SREBP1 protein was accelerated in 97H cells pretreated with CHX knockdown of CREB3L2, while the half-life of SREBP1 protein was extended in Huh7 cells overexpressing CREB3L2, compared to their respective controls, which reinforces that CREB3L2 can stabilize SREBP1 protein (Fig. 5E). The primary pathways for protein degradation encompass the ubiquitin-proteasome system and the autophagy-lysosome pathway. We used the proteasome inhibitor MG132 to treat the 97H cells, knocking down CREB3L2 and showed that the use of MG132 rescued the expression of SREBP1 in 97H cells (Fig. 5F), whereas the addition of an autophagy inhibitor did not have this effect (Supplementary Fig. S4B). Moreover, dysregulation of CREB3L2 has a significant impact on the ubiquitination of SREBP1 protein in 97H and Huh7 cells (Fig. 5G). Altogether, these results suggest that CREB3L2 interacts with SREBP1, enhancing SREBP1 protein expression by limiting SREBP1 ubiquitination and degradation.

Prior research has likewise validated that specific modifications to oncoproteins, including acetylation, are capable of augmenting their own protein stability, thus further facilitating the advancement and differentiation of cancer. Then, we found that changes in CREB3L2 expression also affect the acetylation level of SREBP1, mass spectrometry analysis revealed three potential acetylation sites on SREBP1, Lys395, Lys757 and Lys1151 (Fig. 5H and Supplementary Fig. S4C–E). In order to further identify the lysine sites on SREBP1 where acetylation modification occurs, we constructed three SREBP1 point mutant plasmids based on the results of mass spectrometry sites, and finally showed that the acetylation of SREBP1 mainly occurs at the Lys395 site (Fig. 5I). Next, in order to search for acetyltransferases affecting the SREBP1 protein, we analyzed immunoprecipitates of SREBP1 and identified histone acetyltransferase 1(HAT1) as a potential key player in modulating the acetylation levels of SREBP1 (Fig. 5J). In addition, we also confirmed the interaction of endogenous HAT1 with SREBP1 interact in 97H and Huh7 cells (Supplementary Fig. S4F), while the reduction of SREBP1 acetylation level was observed upon down-regulation of HAT1, and this effect was reversed by co-transfection of simulated acetylated K395Q and shHAT1 plasmids (Fig. 5K), these findings strongly suggest that HAT1 exerts its influence on the acetylation level of SREBP1 through the Lys395 site. Subsequent Co-IP demonstrated that changes in CREB3L2 expression affected the binding capacity of HAT1 and SREBP1, which explains the alteration of SREBP1 acetylation levels induced by CREB3L2 (Fig. 5L). Eventually, ubiquitination analysis and CHX assay showed that the acetylated mimetic K395Q mutant protein reversed changes in the stability and ubiquitination levels of SREBP1 protein (Fig. 5M, Supplementary Fig. S4G, H). The above results suggest that CREB3L2 affects HAT1-mediated acetylation of SREBP1 at K395, which may lead to a reduced level of ubiquitination and increased protein stability of SREBP1.

### SREBP1 is a functionally important target gene for CREB3L2-mediated biological behavior in HCC

**Fig. 6:**
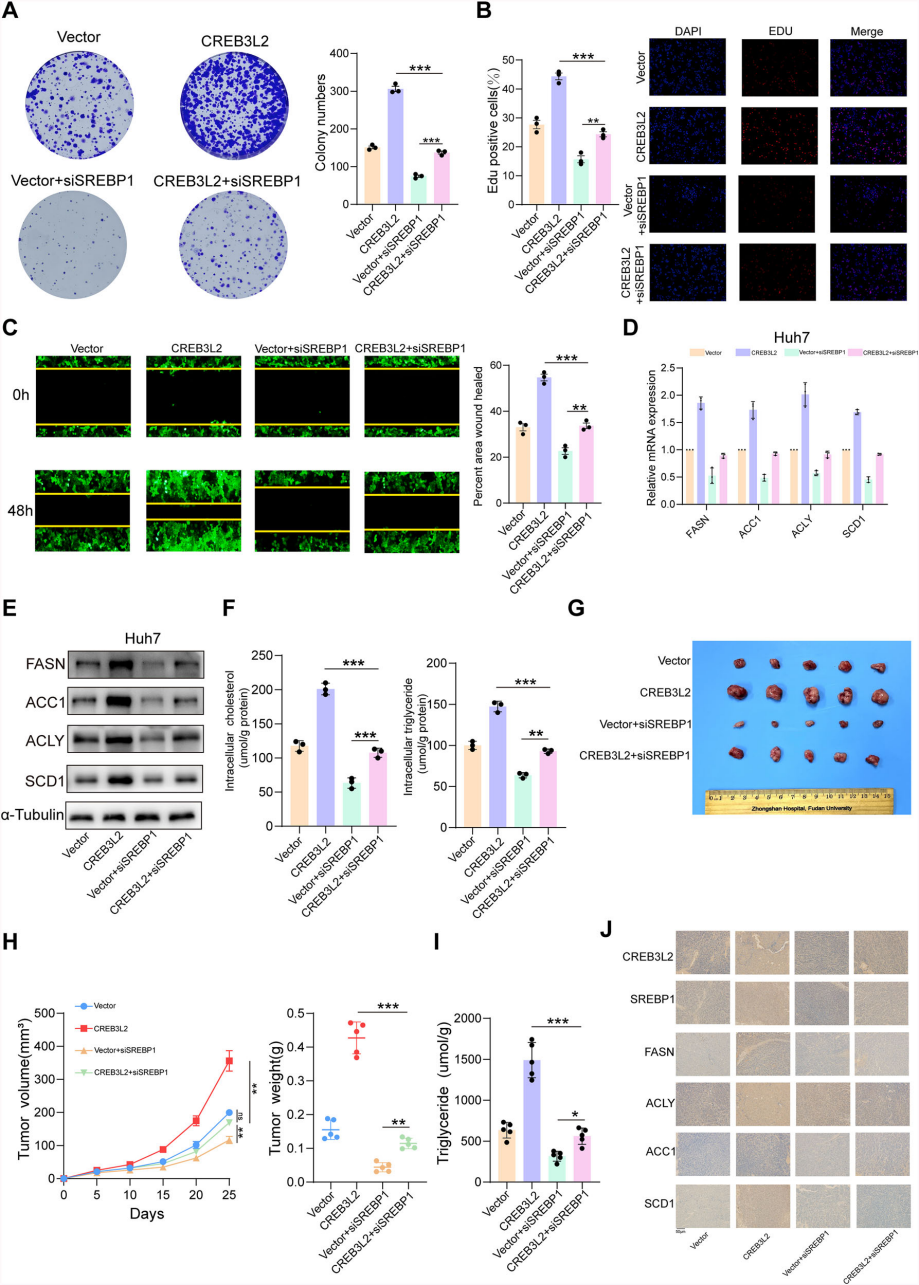
SREBP1 is a functionally important target gene for CREB3L2-mediated biological behavior in HCC. **A**, **B** CCK-8 and EdU proliferation assays conducted in Huh7 cells with CREB3L2 overexpression or SREBP1 knockdown. **C** Wound healing assays conducted after CREB3L2 overexpression or SREBP1 knockdown. **D**, **E** Assess the changes in mRNA and protein expression levels of FASN, ACC1, ACLY, and SCD1 in Huh7 cells. **F** Alterations in triglyceride and cholesterol content in response to CREB3L2 overexpression or SREBP1 knockdown. **G**, **H** Xenograft tumor growth was assessed following CREB3L2 overexpression or SREBP1 knockdown. **I** Measurement of triglyceride levels in harvested xenograft tumors. **J** IHC analysis of CREB3L2, SREBP1, FASN, ACLY, ACC1, and SCD1 in various tumor subgroups. *P < 0.05; **P < 0.01; ***P < 0.001.

Considering the crucial part that lipid metabolism plays in tumor progression, we performed rescue experiments to assess whether SREBP1 is involved in CREB3L2-mediated malignant biological behavior of HCC cells. Colony formation and EDU assays showed that SREBP1 knockdown rescued the increased cell proliferation and colony formation induced by CREB3L2 overexpression in Huh7 cells (Fig. 6A, B). At the same time, silencing SREBP1 effectively reversed the enhanced migration capacity of Huh7 cells induced by CREB3L2 overexpression (Fig. 6C). Importantly, this silencing also rescued the elevated levels of key enzymes involved in fatty acid synthesis that were associated with CREB3L2 overexpression (Fig. 6D, E), and there was a corresponding reversal of triglyceride and cholesterol levels in HCC cells (Fig. 6F).

Additionally, the subcutaneous xenograft model demonstrated that inhibiting SREBP1 significantly suppressed tumor growth driven by CREB3L2 overexpression (Fig. 6G, H). In tumors featuring stable overexpression of CREB3L2, the elevation in triglyceride content can be reversed through the downregulation of SREBP1 as well (Fig. 6I). Immunohistochemical staining demonstrated that within the tumor tissues showing CREB3L2 overexpression, the expressions of SREBP1 and fatty acid-synthesizing enzymes were elevated correspondingly, which is consistent with our in vitro results. Whereas, when cells were co-transfected with lentivirus overexpressing CREB3L2 and downregulating SREBP1, the above effects could be counteracted (Fig. 6J). Thus, the above results indicate that CREB3L2 is capable of regulating lipid metabolism via SREBP1, thereby participating in the growth and metastasis of HCC cells.

### Targeting CREB3L2 reverses lenvatinib resistance in HCC

Drug therapy is essential in the management of intermediate and advanced HCC, with targeted drug therapy playing a crucial role. While lenvatinib has been approved as the first-line therapeutic option for advanced HCC, its overall efficacy rate is only 24.1% [19]. Previous studies have shown that lenvatinib resistance is associated with dysregulated lipid metabolism in HCC cells [20]. In addition, HCC cells with elevated levels of CREB3L2 exhibit heightened lipid synthesis, contributing to the advancement of tumors. As a result, we postulate that CREB3L2 may play a role in inherent resistance to lenvatinib.

**Fig. 7:**
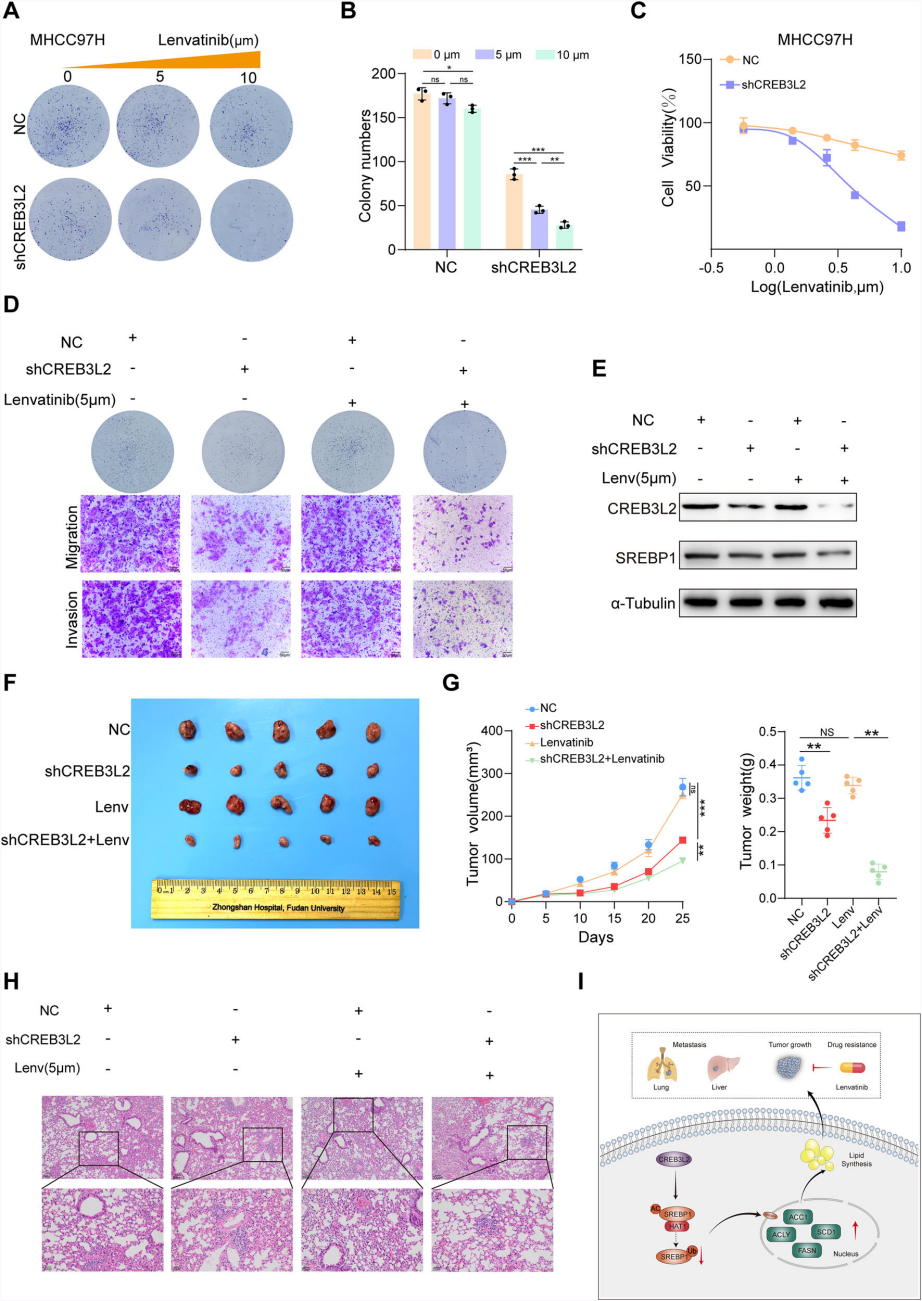
Targeting CREB3L2 reverses lenvatinib resistance in HCC. **A**, **B** Silencing CREB3L2 in 97H cells significantly increases sensitivity to lenvatinib. **C** Determination of cell viability in 97H cells treated with lenvatinib using the CCK-8 assay. **D** Suppression of CREB3L2 expression markedly enhances lenvatinib-mediated inhibition of tumor cell malignant phenotypes. **E** Knockdown of CREB3L2 enhances the inhibitory effect of lenvatinib on SREBP1 expression. **F**, **G** Xenograft tumor growth of 97H cells with CREB3L2 knockdown treated with lenvatinib. **H** Representative lung metastasis sections from different treatment groups of nude mice. **I** A simple mechanistic diagram for the entire text. *P < 0.05; **P < 0.01; ***P < 0.001.

In an attempt to validate the aforementioned theoretical hypothesis, we explored the cytotoxic and therapeutic effects of lenvatinib on HCC cells with CREB3L2 knockdown or overexpression, and the effect of different concentrations of lenvatinib was assessed in primary resistant cell line MHCC97H or sensitive cell line Huh7 [21]. The findings revealed that HCC cells with relatively high levels of CREB3L2 demonstrated marked resistance to lenvatinib, while downregulation of CREB3L2 notably enhanced the sensitivity to lenvatinib (Fig. 7A–C and Supplementary Fig. S5A–C). Furthermore, we found that changes in CREB3L2 expression levels not only affected the proliferation of HCC cells but also significantly altered their resistance to lenvatinib. Transwell migration and matrix gel invasion assays demonstrated that suppression of CREB3L2 reduced cell migration and invasion, ultimately restoring the anti-tumor effects of lenvatinib in vitro (Fig. 7D and Supplementary Fig. S5D, E). Conversely, overexpression of CREB3L2 enhanced cell migration and invasion capabilities and increased resistance to lenvatinib (Supplementary Fig. S5F–H). Deeply, the reduction in HCC cancerous biological behavior following downregulation of CREB3L2 can be attributed to a significant decrease in SREBP1 expression. Despite lenvatinib’s partial inhibition of SREBP1 expression, it fails to efficiently suppress the tumor progression in HCC owing to the comparatively high expression level of CREB3L2. Therefore, the downregulation of SREBP1 induced by the knockdown of CREB3L2 successfully overcomes lenvatinib resistance in HCC cells via the suppression of lipid metabolism (Fig. 7E). Consistent with expectations, within the tumor xenograft model of 97H cells subjected to shCREB3L2 treatment, it was observed that the knockdown of CREB3L2 substantially inhibited tumor growth and reinstated the anti-tumor effectiveness of lenvatinib (Fig. 7F, G). Subsequently, downregulation of CREB3L2 reduced lung metastatic lesions and greatly attenuated lenvatinib resistance to tumor metastasis (Fig. 7H and Supplementary Fig. S5I). The results stated above imply that CREB3L2 is capable of engaging in HCC progression and lenvatinib resistance through affecting SREBP1-mediated lipid metabolism (Fig. 7I).

## Discussion

Hepatocellular carcinoma presents a substantial challenge to patient survival as a result of its elevated morbidity and mortality rates [22]. The prognosis for patients with HCC is unfavorable due to the advanced stage of the disease at diagnosis, as well as the elevated incidence of recurrence or metastasis at an advanced stage, accompanied by a notable degree of tolerance to systemic therapy [23]. In both in vitro and in vivo experiments, we demonstrated that the CREB3L2/HAT1/SREBP1 axis promotes tumor cell proliferation and metastasis by enhancing lipid metabolism, and targeting CREB3L2 can partially overcome lenvatinib resistance to augment therapeutic efficacy.

It is widely known that the liver serves as a crucial organ involved in protein, sugar, and lipid metabolism [24]. Even in the presence of adequate external fatty acids, HCC cells still depend on de novo synthesis for their own supply of fatty acids [25–27]. This indicates that exploring abnormal lipid metabolic pathways can offer a more thorough insight into the mechanisms of HCC progression. Aberrant expression of fatty acid synthase has been documented in various tumors, including HCC. Our findings indicate that CREB3L2 can enhance lipogenesis in HCC cells by up-regulating the key enzyme involved in fatty acid synthesis, which energizes the malignant proliferation and metastasis of tumor cells.

Previously recognized as an oncogene, SREBP1 initially binds to the endoplasmic reticulum membrane in its inactive form, undergoes cleavage by hydrolysis, translocates into the nucleus as an active entity, and subsequently promotes lipid biosynthesis [28–30]. We initially demonstrated that CREB3L2 inhibits the degradation of SREBP1 protein, thereby enhancing its protein stability. Acetylation modification of lysine can impact the stability and activity of oncogenic proteins [31–34]. Subsequently, we identified HAT1 as capable of modifying SREBP1 at Lys395, and found that CREB3L2 also enhances the binding efficiency of HAT1 and SREBP1. Although we have discovered the impact of the CREB3L2/HAT1/SREBP1 regulatory axis on the progression of HCC, further analysis is required to elucidate the specific molecular mechanisms underlying this interaction.

Currently, there is a lack of effective small-molecule inhibitors capable of suppressing CREB3L2 expression. However, siRNA delivery systems (lipid nanoparticles or GalNAc conjugates) have achieved clinical success in liver diseases [35, 36]. These efficient liver-targeted delivery modalities can selectively silence the target gene CREB3L2, and their combination with lenvatinib may yield superior therapeutic efficacy. Additionally, Proteolysis-targeting chimeras (PROTACs) can simultaneously bind the target protein and E3 ubiquitin ligases, thereby bringing the target protein into proximity with the E3 ligase and inducing ubiquitination of the target protein [37]. To achieve sustained degradation, a PROTAC targeting CREB3L2 could exploit its protein interactions with HAT1/SREBP1 to direct degradation. Given the pronounced heterogeneity among HCC patients, we should pursue more clinical-translational studies on CREB3L2 in future work, which is essential for identifying more effective strategies to overcome drug resistance.

Collectively, our research findings indicate that the CREB3L2/HAT1/SREBP1 axis promotes lenvatinib resistance and the progression of HCC by affecting lipid metabolism. Furthermore, targeting CREB3L2 may enhance the efficacy of lenvatinib treatment for HCC.

## Supplementary information

Supplementary material

Original Western blots

## Data Availability

The data that support the results of this study are available from the corresponding authors upon reasonable request.

## Material availability

The data that support the results of this study are available from the corresponding authors upon reasonable request.
