# Supplementary material for: Targeting CREB3L2-mediated lipid metabolism overcomes lenvatinib resistance and attenuates the progression of hepatocellular carcinoma
Source: Cell Death Dis. 2025 Nov 24;16(1):855. doi: 10.1038/s41419-025-08250-3 (PMC12644697; doi:10.1038/s41419-025-08250-3)
Supplement: Supplementary file 1 — Supplementary material [file 41419_2025_8250_MOESM1_ESM.pdf]

Supplementary Figures

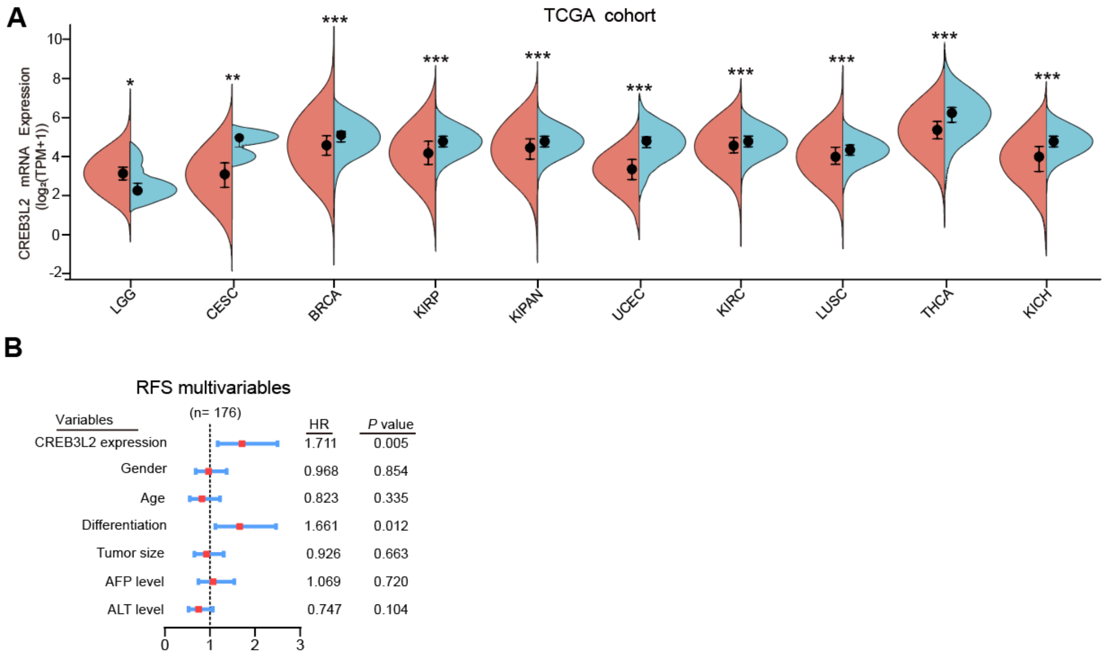

Figure S1 CREB3L2 is upregulated and correlates with poor prognosis in HCC.

(A) Relative expression of CREB3L2 in brain Lower Grade Glioma(LGG), cervical squamous cell carcinoma and endocervical adenocarcinoma(CESC), breast invasive carcinoma(BRCA), kidney renal papillary cell carcinoma(KIRP), pan-kidney cohort (KIPAN), uterine Corpus Endometrial Carcinoma(UCEC), kidney renal clear cell carcinoma(KIPC), lung squamous cell carcinoma(LUSC), thyroid carcinoma(THCA), kidney Chromophobe(KICH).**(B)** Multivariable Cox analysis of clinical prognostic parameters for RFS. *,P < 0.05; **, P < 0.01; ***, P < 0.001.

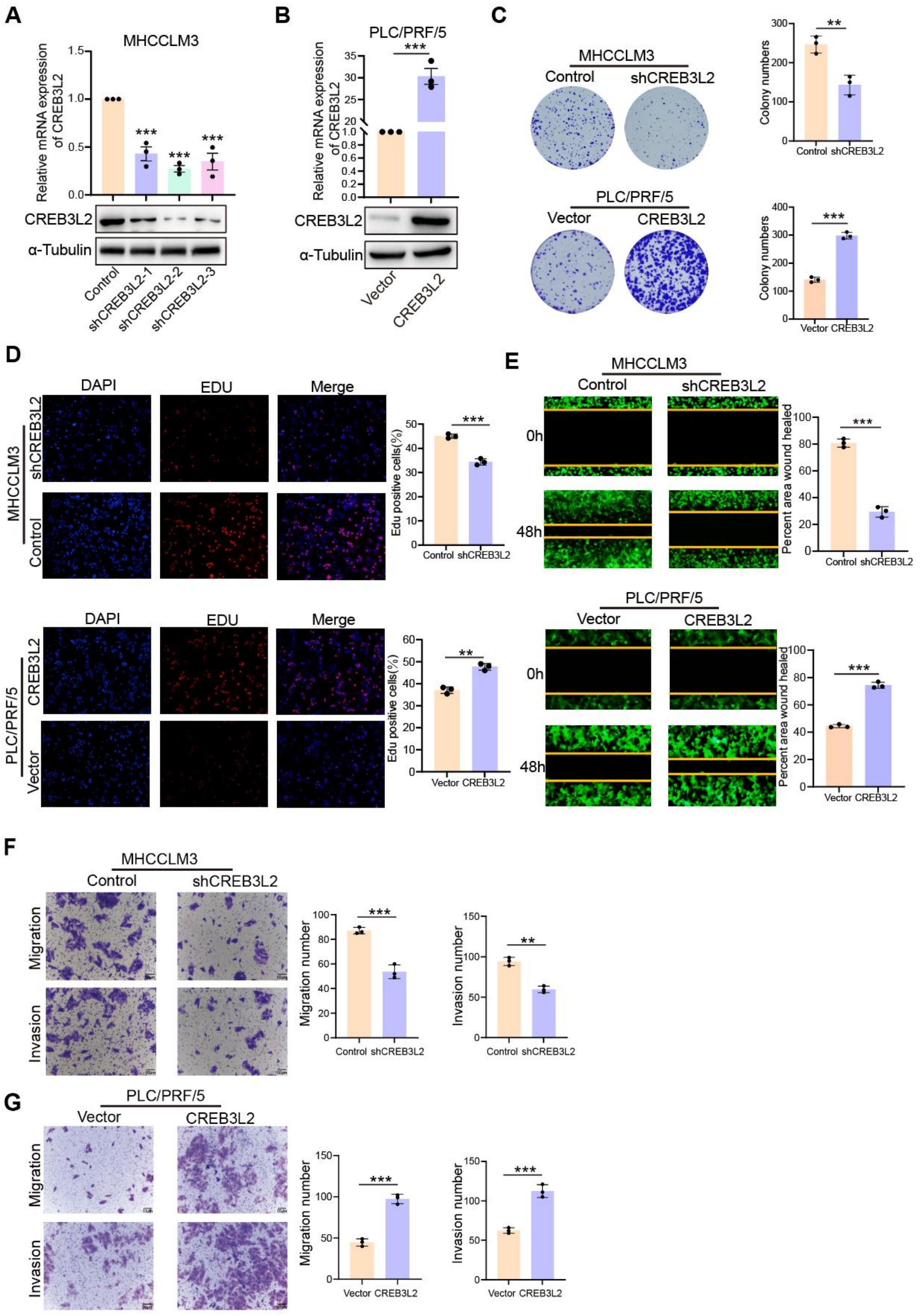

FigureS2 CREB3L2 facilitates HCC cell proliferation and metastasis in vitro

The transfection efficiency of knockdown and overexpression of CREB3L2 in LM3 and PLC cells was confirmed through qPCR **(A)** and Western blotting **(B)**. **(C-D)** Colony formation and EDU assays were performed after CREB3L2 was knocked down or overexpressed in LM3 and

PLC cells. **(E-G)** After knockdown or overexpression of CREB3L2, the migration and invasion capabilities of HCC cells were assessed using scratch and transwell assays. *,P < 0.05; **, P < 0.01; ***, P < 0.001.

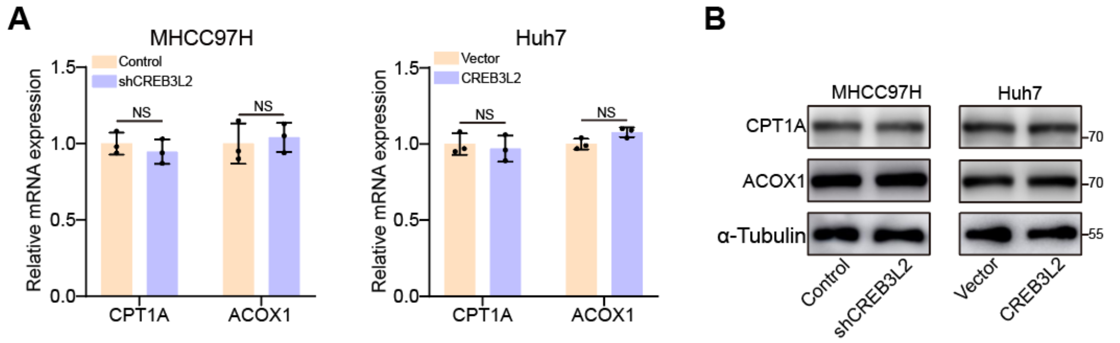

FigureS3 CREB3L2 modulates fatty acid metabolism pathways

(A-B)After knocking down or overexpressing CREB3L2, assess the alteration of CPTIA and ACOX1.

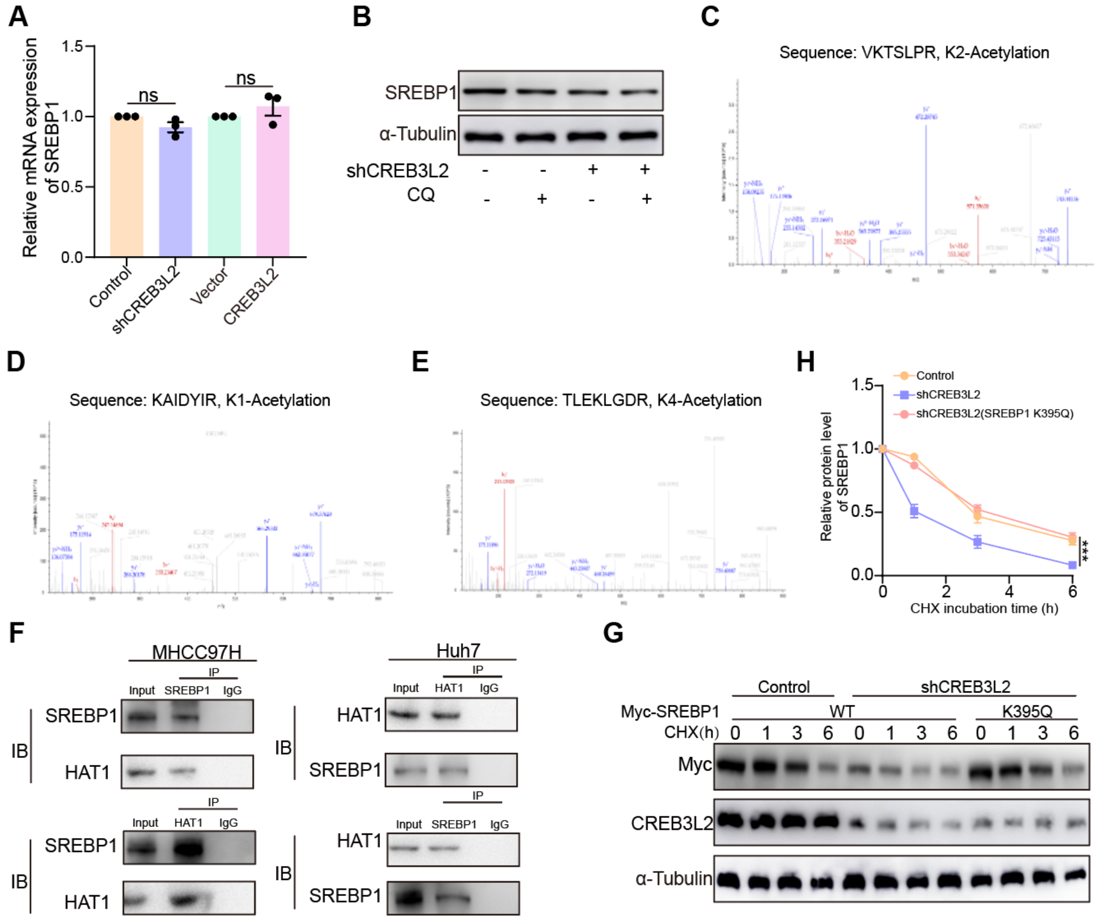

FigureS4 CREB3L2 attenuates ubiquitinated degradation of SREBP1 protein by enhancing HAT1-mediated acetylation

(A) The effect of CREB3L2 on SREBP1 mRNA levels in HCC cells.(B) The protein expression level of SREBP1 in 97H-shCREB3L2 cells in the presence of CQ. (C-E) Prediction of acetylation sites on SREBP1 through mass spectrometry analysis.(F) Co-IP demonstrated the combination of HAT1 and SREBP1 in Huh7 cells.(G-H) Western blot analysis of the effect of CREB3L2 and SREBP1(K395Q) on the half-life of SREBP1 in HCC cells treated with cycloheximide (CHX).

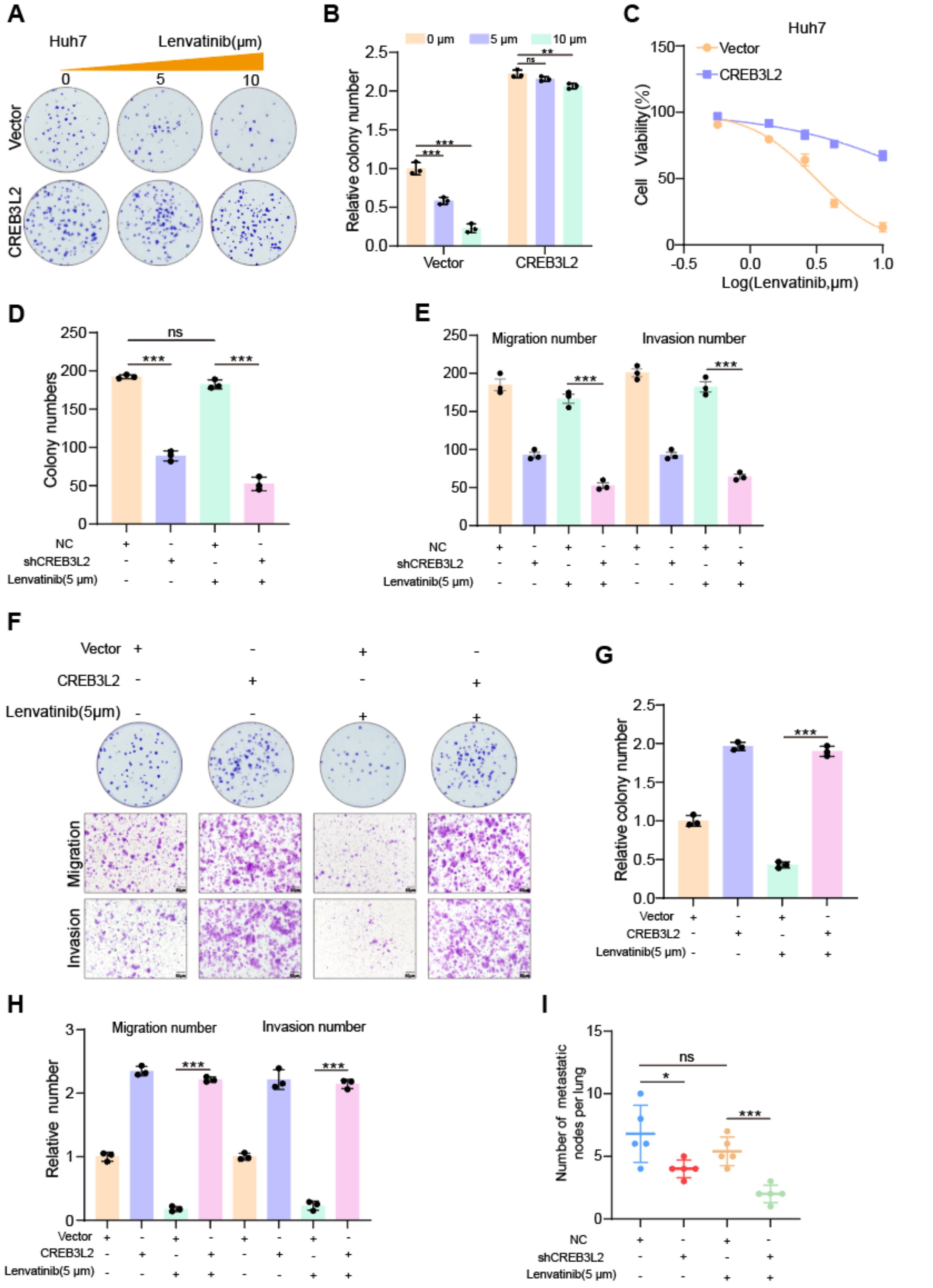

FigureS5 Targeting CREB3L2 reverses lenvatinib resistance in HCC

(**A-B**) Overexpression of CREB3L2 in Huh7 cells can significantly increase resistance to lenvatinib.(**C**) Determination of cell viability in Huh7 cells treated with lenvatinib using the CCK-8 assay **(D-E)** Inhibition of CREB3L2 expression markedly enhances the effects of lenvatinib on cell proliferation, migration, and invasion.(**F-H**) Overexpression of CREB3L2

can significantly weaken the inhibition of the malignant phenotype of tumor cells mediated by lenvatinib.**(I)** The specific differences regarding the metastatic pulmonary nodules among distinct groups.

Supplemental Table-1 Relationships between CREB3L2 and clinicopathological characteristics in the Zhongshan cohort

Variables	Total (n=176)	CREB3L2 low (n=82)	CREB3L2 high (n=94)	χ2	*P*
Age				1.200	0.273
≤50	76(43.2)	39	37		
>50	100(56.8)	43	57		
AFP				1.354	0.245
≤20	65(36.9)	34	31		
>20	111(63.1)	48	63		
CEA				0.085	0.770
≤5	162(92.0)	76	86		
>5	14(8.0)	6	8		
CA199				0.743	0.389
≤36	134(76.1)	60	74		
>36	42(23.9)	22	20		
Cirrhosis				0.368	0.544
No	29(16.5)	15	14		
Yes	147(83.5)	67	80		
Tumor size					
≤5	85(48.3)	40	45	0.014	0.904
>5	91(51.7)	42	49		
Differentiation					
I/II	111(63.1)	43	68	7.447	0.006
III/IV	65(36.9)	39	26		
Child grade					
A	166(94.3)	77	89	0.050	0.804
B/C	10(5.7)	5	5		
MVI					
Negative	100(56.8)	43	57	1.200	0.273
Positive	76(43.2)	39	37		
BCLC stage					
0/A	87(49.4)	48	39	5.092	0.024
B/C	89(50.6)	34	55		
ALT					
≤40	102(58.0)	50	52	0.545	0.448
>40	74(42.0)	32	42		
AST					
≤37	124(70.5)	62	62	1.686	0.194
>37	52(29.5)	20	32		

Supplemental Table-2 Reagents and antibodies

Antibody	Vendor	catalog number
CREB3L2	Santa Cruz	sc-515816
SREBP1	Proteintech	14088-1-AP
	/HUABIO	ER1917-19
α-Tubulin	Abclonal	A6830
HAT1	Proteintech	11432-1-AP
FASN	Proteintech	10624-2-AP
ACC1	Proteintech	21923-1-AP
ACLY	Proteintech	15421-1-AP
SCD1	Proteintech	28678-1-AP
Ubiquitin	Cell Signaling Technology	#20326
Ac-lysine	Santa Cruz	sc-32268
MG132	MCE	HY-13259
Cycloheximide	MCE	HY-12320
Lenvatinib	Selleck	#S1164

Supplementary materials and methods

5-ethynyl-2′-deoxyuridine（Edu） and clone formation assay

The Edu kit was procured from Beyotime Biosciences Inc (Shanghai, China), and all experimental procedures were conducted step by step according to the instructions provided in the kit's manual, and samples were ultimately photographed under a fluorescence microscope for record-keeping. In the clone formation experiments, an equal number of cells (1×10^3 cells/well) were seeded and the medium was refreshed every fourth day. After two weeks, rinsed each well twice with 1ml PBS, fixed in 4% paraformaldehyde for 10-15 minutes, stained with 1% crystal violet for 15 minutes, washed with PBS and air-dried finally. After photographing the plates, the number of cell colonies was quantified.

Would healing assay

Cells were seeded into six-well plates and three cell scratches were made using a 10ul pipette tip the next day. The cell clusters were removed by washing with PBS, followed by changing to serum-free medium and continued incubation for 24-48 hours. Cell migration distance was captured using a microscope, and the cell migration rate was computed.

Migration and invasion assay

Transwell chambers with or without Matrigel (Corning, USA) were utilized to assess the invasive and migratory capabilities of HCC cells. The specified number of cells (2×10^4 for invading cells and 1.5×10^4 for migrating cells) were seeded in the upper chamber containing DMEM, while the lower chamber was filled with DMEM supplemented with 10% fetal bovine serum. After 24-48 hours, the cells were rinsed with PBS, fixed in 4% paraformaldehyde for 10 minutes, and stained with crystal violet. Afterward, untranslocated cells on the upper surface were removed with a cotton swab, and the chambers were placed under an inverted microscope

to observe and photograph the migrated cells at a magnification of 20-40x.

Oil red O staining

HCC cells were seeded onto 12-well plates pre-coated with sterile coverslips and incubated overnight. The cells were then rinsed with fresh PBS and fixed with 4% paraformaldehyde. Afterwards, the cells on the plates were stained with an appropriate amount of Oil Red O solution (Beyotime, Shanghai, China) and allowed to sit for 30 minutes at room temperature. The nuclei were counterstained with hematoxylin staining solution (Beyotime, Shanghai, China). Finally, the average cell staining intensity was evaluated using ImageJ software, and the quantified values represent the amount of oil in the cells, indicating the level of lipid accumulation.

Determination of cholesterol and triglycerides

Triglycerides and cholesterol were quantified in the hepatocellular carcinoma cells and tissues using the Triglyceride and Cholesterol Assay Kit（Applygen，Beijing，China） following standard protocols. Standard curves were plotted to calculate the corresponding concentrations and then corrected according to the protein content.

Experimental grouping

Mice were selected independently of weight and health status. They were randomly assigned to groups using sealed containers. The experimental group received specific interventions, while the control group was treated with doses and administration methods identical to those of the experimental group.

If a sample's value significantly deviates from the mean of its experimental group, it will either be excluded or the experiment for that group will be repeated to ensure data accuracy.

Animal experiments

All 6-week-old female nude mice were purchased from Jiesijie company(Shanghai,China). Upon arrival, the mice were randomly assigned into groups, with 5 mice per group.
