# Supplementary material for: Targeting CREB3L2-mediated lipid metabolism overcomes lenvatinib resistance and attenuates the progression of hepatocellular carcinoma
Source: Cell Death Dis. 2025 Nov 24;16(1):855. doi: 10.1038/s41419-025-08250-3 (PMC12644697; doi:10.1038/s41419-025-08250-3)

Fig 1E

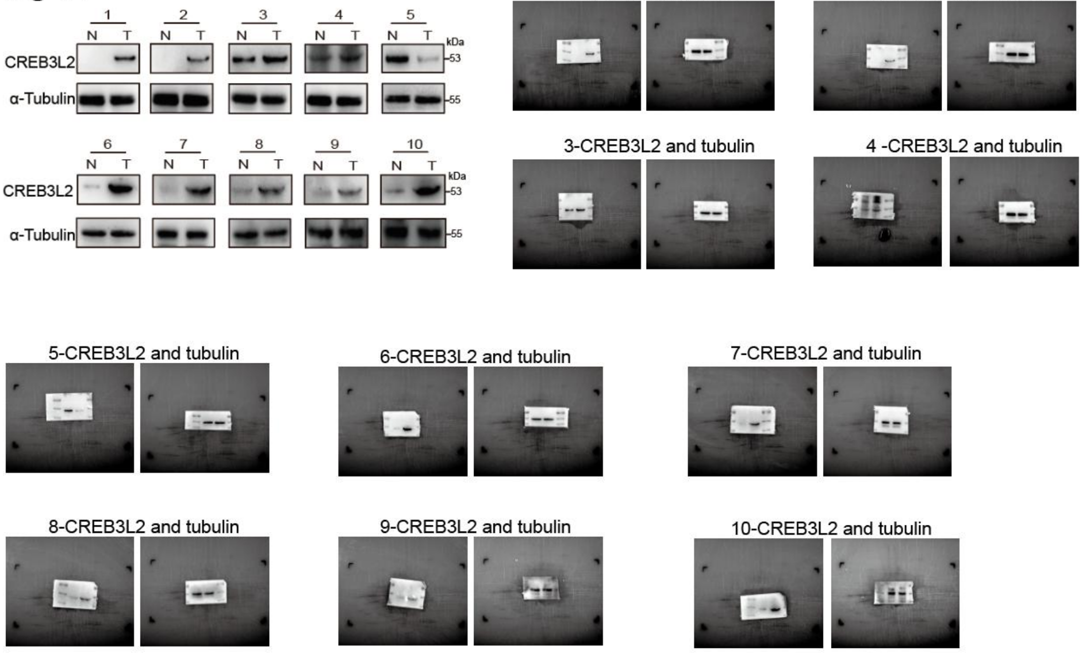

Fig 2B

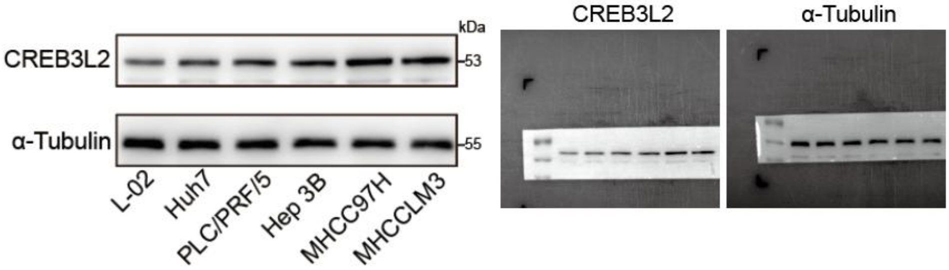

Fig 2C

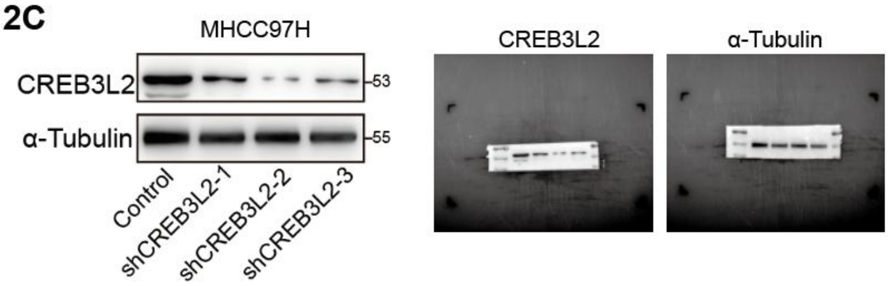

Fig 2D

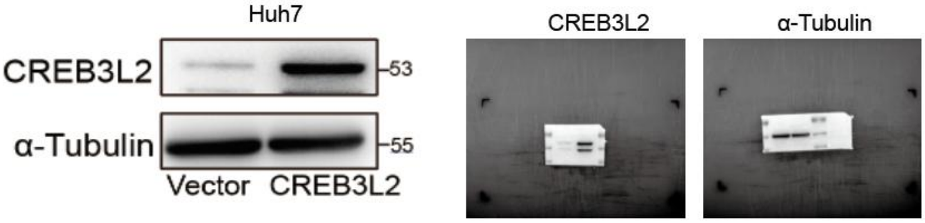

Fig 4I

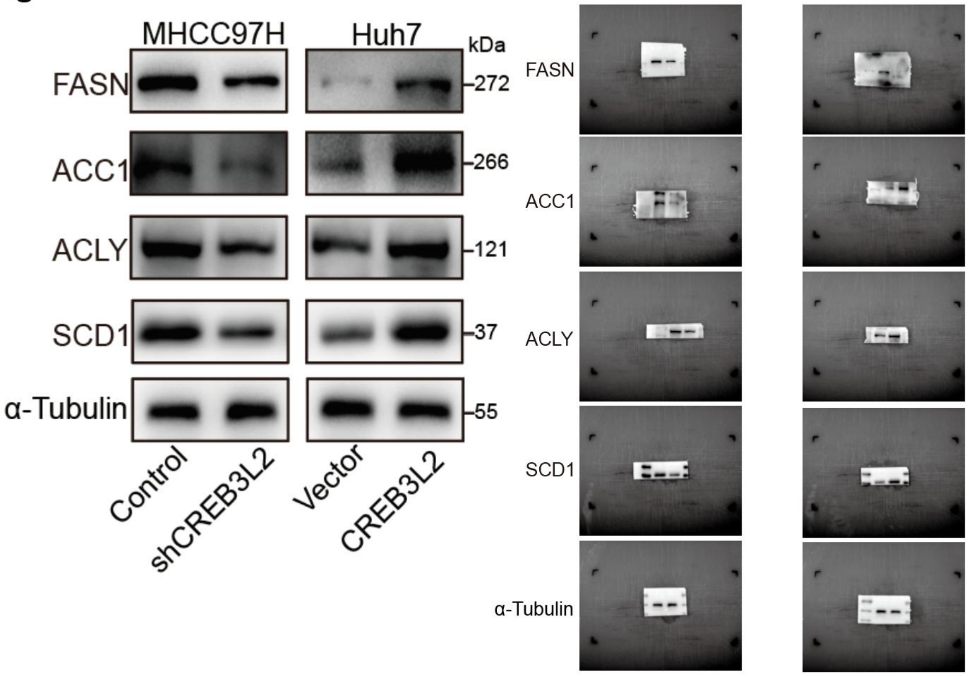

Fig 5B

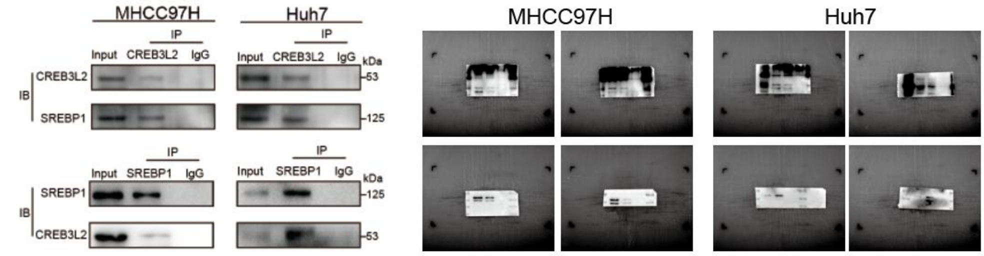

Fig 5D

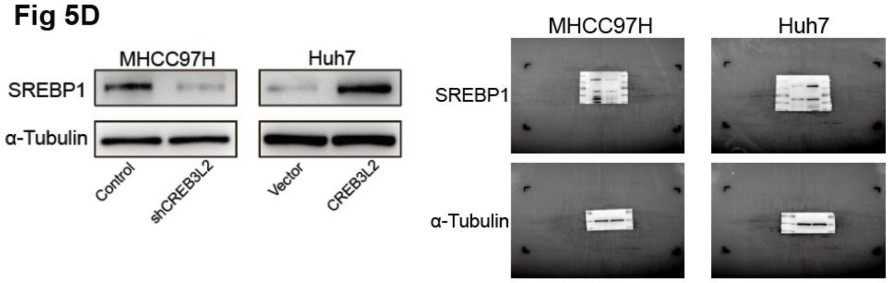

Fig 5E

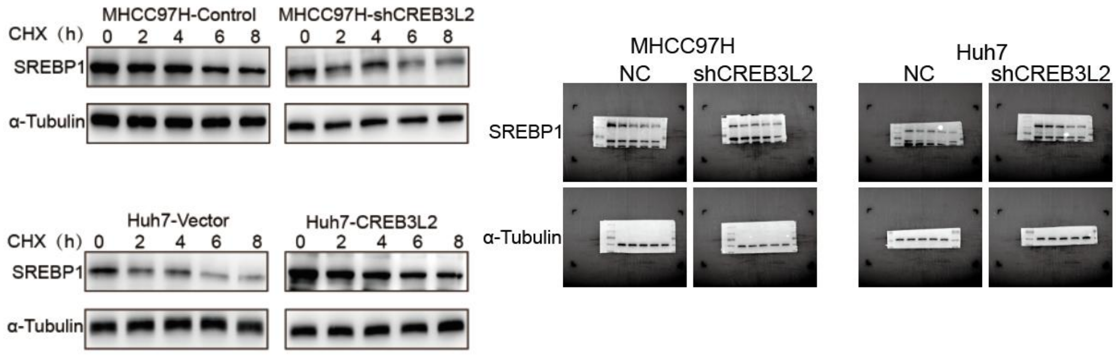

Fig 5F

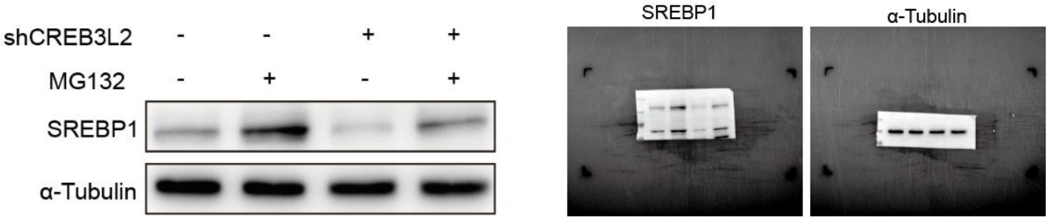

Fig 5G

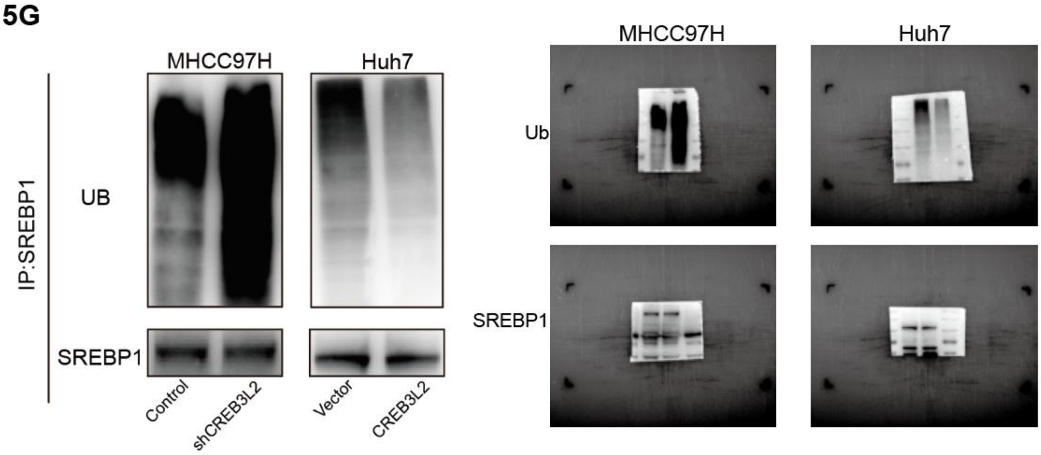

Fig 5H

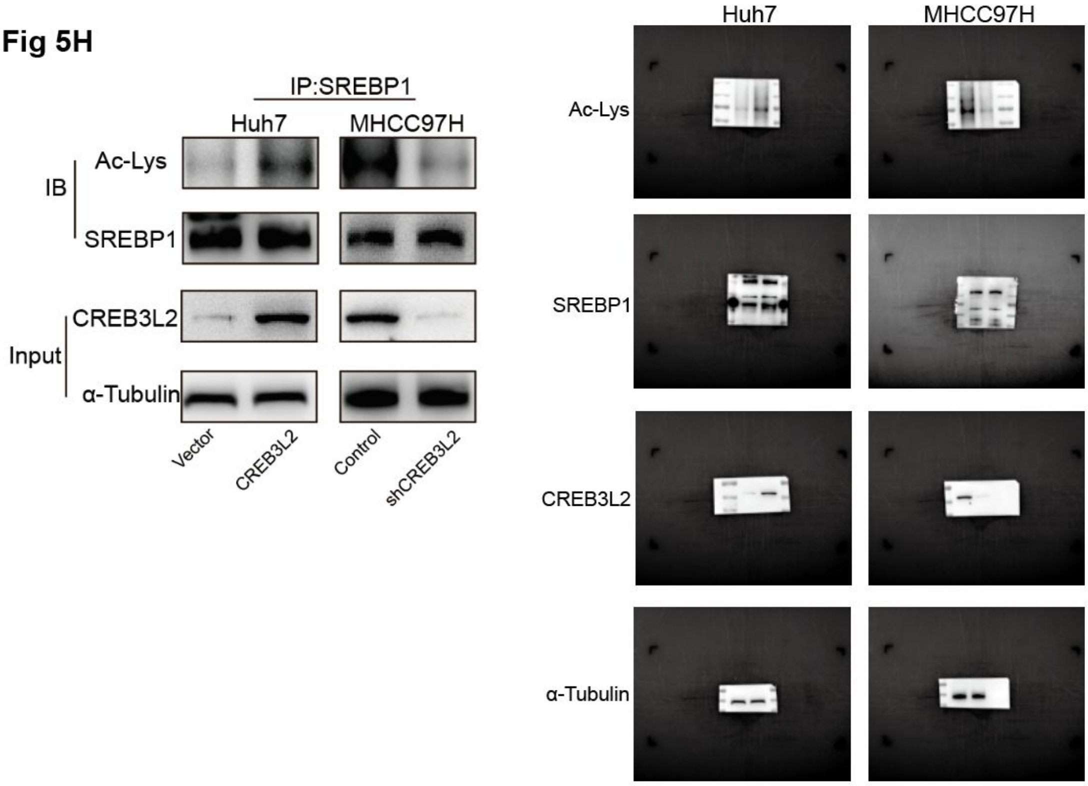

Fig 5I

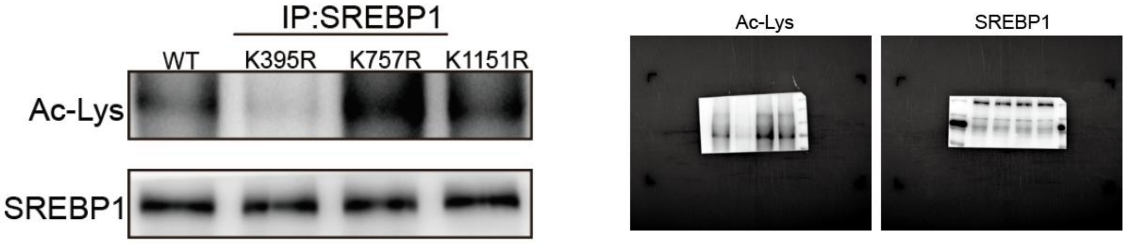

Fig 5K-L

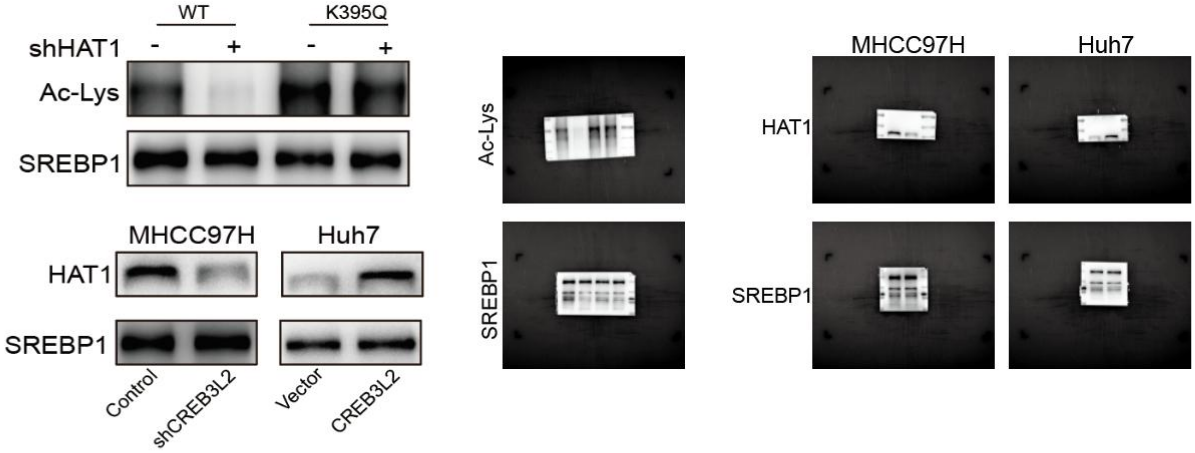

Fig 5M

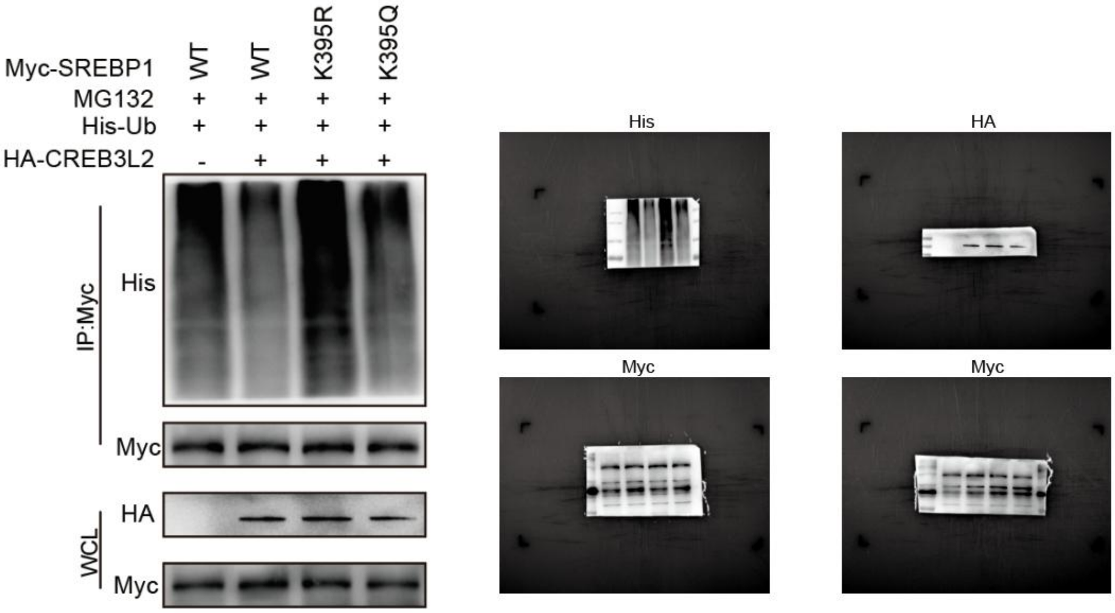

Fig 6E

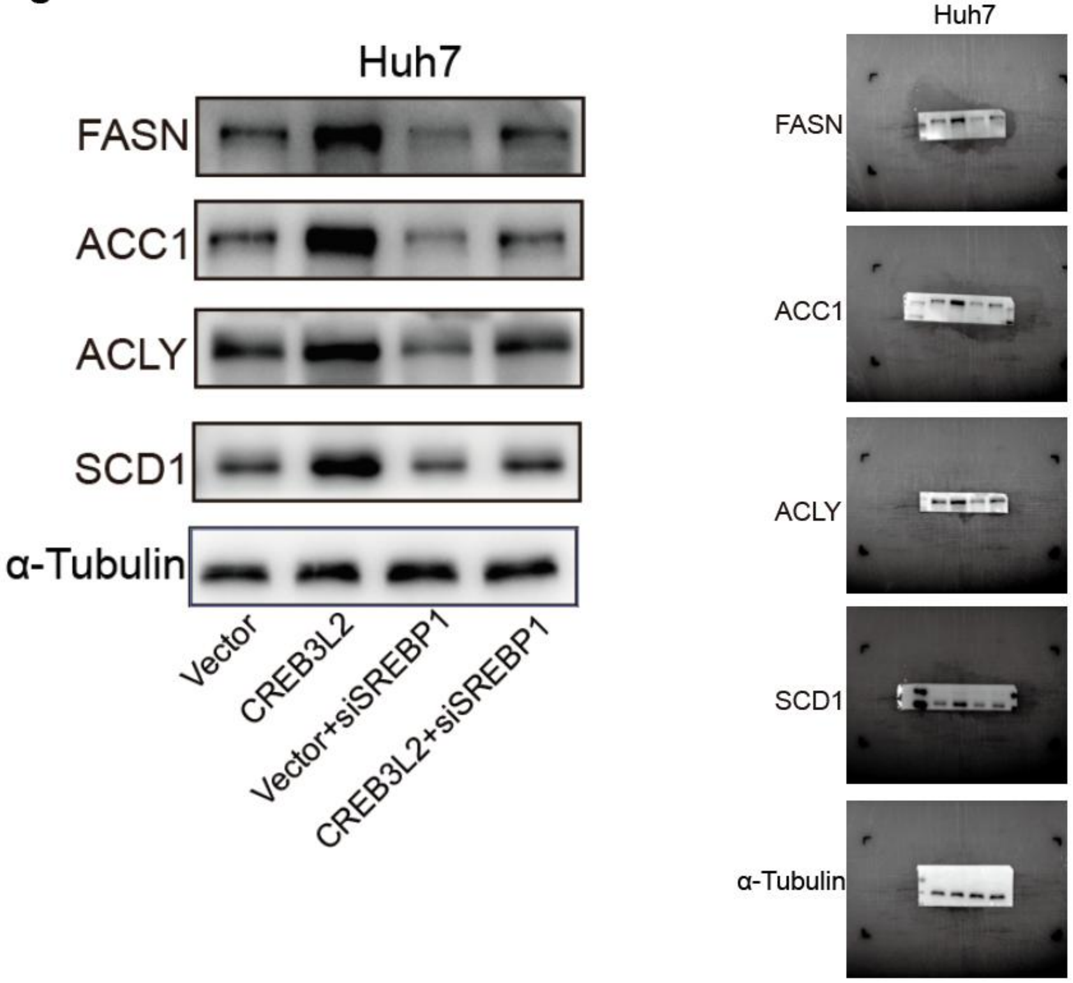
Huh7
FASN
ACC1
ACLY
SCD1
α-Tubulin
Vector
CREB3L2
Vector+siSREBP1
CREB3L2+siSREBP1
Huh7
FASN
ACC1
ACLY
SCD1
α-Tubulin

Fig 7E

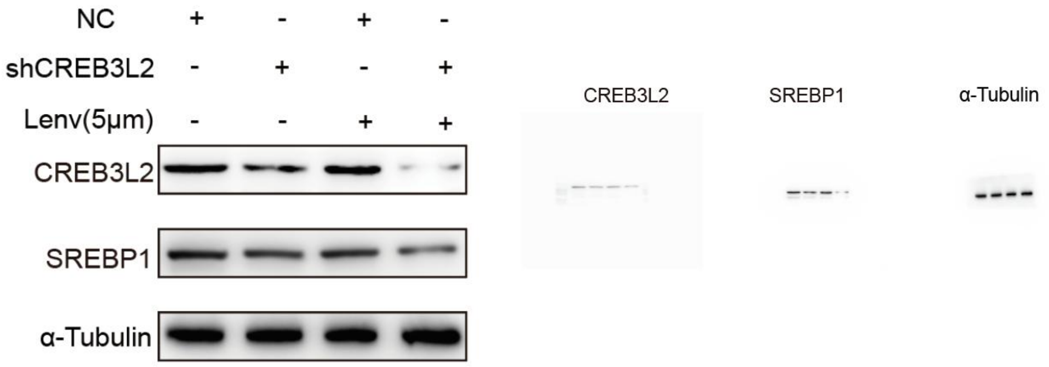
NC + - + -
shCREB3L2 - + - +
Lenv(5μm) - - + +
CREB3L2
SREBP1
α-Tubulin
CREB3L2
SREBP1
α-Tubulin

FigS3B

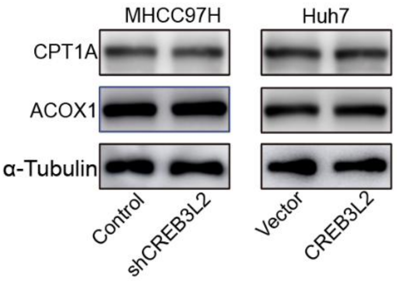

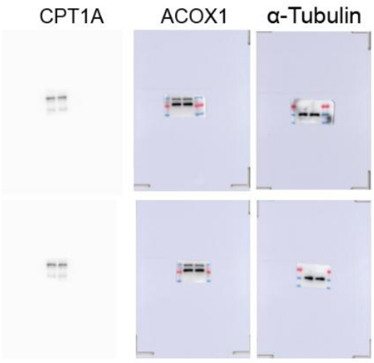

FigS4B

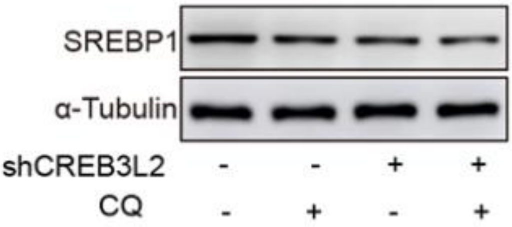

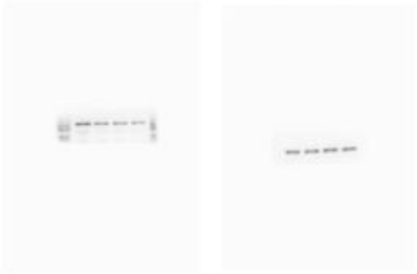

FigS4F

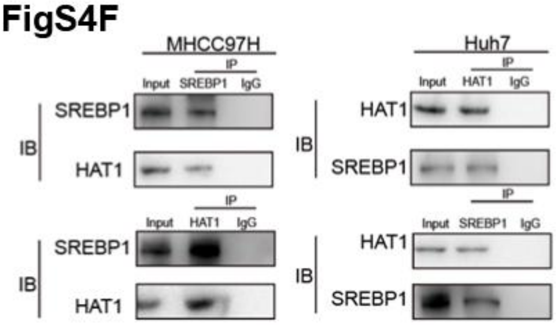

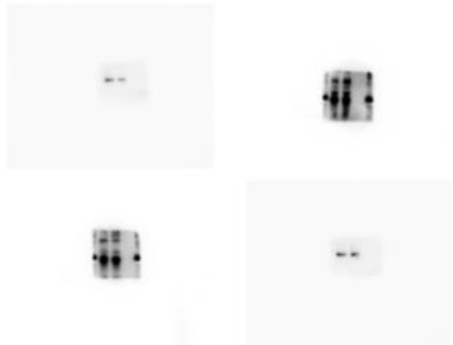

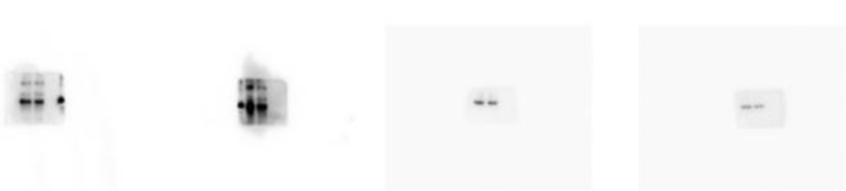

FigS4G

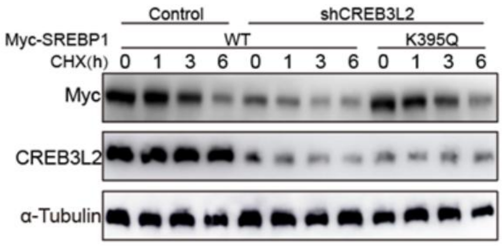

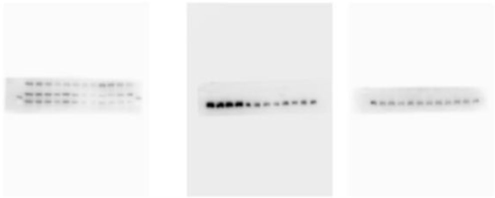

Supplement: Supplementary file 2 — Original Western blots [file 41419_2025_8250_MOESM2_ESM.pdf]
